# Mineral weathering is linked to microbial priming in the critical zone

**DOI:** 10.1038/s41467-022-35671-x

**Published:** 2023-01-20

**Authors:** Qian Fang, Anhuai Lu, Hanlie Hong, Yakov Kuzyakov, Thomas J. Algeo, Lulu Zhao, Yaniv Olshansky, Bryan Moravec, Danielle M. Barrientes, Jon Chorover

**Affiliations:** 1 School of Earth and Space Sciences, Peking University, 100871 Beijing, China; 2 Department of Environmental Science, University of Arizona, Tucson, AZ 85721-0038 USA; 3 State Key Laboratory of Biogeology and Environmental Geology, School of Earth Sciences, China University of Geosciences, 430074 Wuhan, China; 4 Department of Soil Sciences of Temperate Ecosystems, Department of Agricultural Soil Sciences, University of Göttingen, 37077 Göttingen, Germany; 5 Peoples Friendship University of Russia (RUDN University), Moscow, Russia; 6 Department of Geosciences, University of Cincinnati, Cincinnati, OH 45221-0013 USA; 7 State Key Laboratory of Geological Processes and Mineral Resources, China University of Geosciences, 430074 Wuhan, China; 8 Department of Crop, Soil and Environmental Sciences, Auburn University, Auburn, AL 36849 USA

**Keywords:** Carbon cycle, Carbon cycle

## Abstract

Decomposition of soil organic matter (SOM) can be stimulated by fresh organic matter input, a phenomenon known as the ‘priming effect’. Despite its global importance, the relationship of the priming effect to mineral weathering and nutrient release remains unclear. Here we show close linkages between mineral weathering in the critical zone and primed decomposition of SOM. Intensified mineral weathering and rock-derived nutrient release are generally coupled with primed SOM decomposition resulting from “triggered” microbial activity. Fluxes of organic matter products decomposed via priming are linearly correlated with weathering congruency. Weathering congruency influences the formation of organo-mineral associations, thereby modulating the accessibility of organic matter to microbial decomposers and, thus, the priming effect. Our study links weathering with primed SOM decomposition, which plays a key role in controlling soil C dynamics in space and time. These connections represent fundamental links between long-term lithogenic element cycling (= weathering) and rapid turnover of carbon and nutrients (= priming) in soil.

## Abstract (web summary)

Mineral weathering and microbial priming are two important processes that regulate soil formation and CO_2_ emissions. Here the authors link weathering with primed organic matter decomposition, which plays a key role in controlling soil C dynamics.

## Introduction

Soils contain more carbon (C) than plant biomass and the atmosphere combined, and the release of this C as CO_2_ would strongly exacerbate global warming^1,2^. Decomposition of organic compounds in soils involves either the hydrolysis of biopolymers and dispersion of supramolecular aggregates into smaller and simpler components such as those found in dissolved organic matter (DOM), or complete mineralization and release of CO_2_ and nutrients^3,4^. These processes contribute to a large CO_2_ efflux from the soil that is a major part of the global C cycle. DOM represents a fundamental link between terrestrial and aquatic C and nutrient cycles and plays an important role in soil C dynamics and biogeochemical processes^5,6^. Though DOM largely comprises byproducts of microbial decomposition of plant materials, it is a mobile energy source for heterotrophs and can fuel microbial activity^7^. Fluxes of DOM in terrestrial ecosystems are several-fold larger than those in riverine systems^8^. To date, studies of DOM concentration and composition have focused mainly on surface waters^9–11^ and water-soluble organic matter in soils^12,13^ [note: when applied to soils, the term “DOM” refers to soluble organic matter extracted in situ by pore water samplers^14^]. The vertical dynamics and environmental controls of DOM in mobile soil porewaters have received insufficient attention, limiting our understanding of the transport, cycling and C-budget consequences in such systems and their responses to environmental change.

Release of root exudates or downward movement of labile C in soils can lead to a ‘priming effect’—that is, a short-term increase in the rate of microbial decomposition of soil organic matter (SOM) resulting from fresh organic C input^15,16^ (Table 1), although field-based observations of the priming effect are scant^17^. Fresh plant-derived DOM transported downward from aboveground and topsoil is an important C source and carrier for soil C dynamics and the priming effect. Due to its crucial role for soil C loss and global C and nutrient cycling, and in feedbacks of ecological processes to climate change, the priming effect has attracted considerable attention in ecosystem, soil, and environmental sciences (e.g., refs. ^16–19^). However, the priming effect has seldom been incorporated into Earth System Models, rendering the robustness of predictions of global soil C stocks and fluxes uncertain^1,19^. Prior studies have identified nutrient availability, soil physico-chemical conditions (e.g., pH, clay-mineral composition, and particle-size distribution), microbial properties (i.e., biomass, activity and community structure), and climate variables as important factors controlling the priming effect^15,20,21^, yet the underlying mechanisms of priming remain controversial. Several studies have inferred that root exudates boost microbial activity and enzyme production and serve as ‘co-metabolites’^22–25^. Keiluweit et al.^22^ proposed a further mechanism, i.e., that root exudates liberate organic matter from protective mineral-organic associations and accelerate their decomposition.

**Table 1:** Terminology and definitions

Terminology	Definition
Priming effect	The change in decomposition rate of soil organic matter after input of fresh organic matter or nutrients into soil. Priming effect refers to both the absolute priming effect and the relative priming effect.
Absolute priming effect	The change in organic matter decomposition rate following addition of a labile substrate in absolute units (e.g., mg C of CO_2_ kg^−1^ soil day^−1^, or g C of CO_2_ m^−2^ soil yr^−1^).
Relative priming effect	The priming effect relative to the organic matter decomposition rate in the “control” soil (i.e., basal respiration rate) – the soil without input of fresh organic matter or nutrients.
Congruent weathering	The weathering process that yields only dissolved phases as reaction products. Taking the weathering of albite by oxalic acid as an example: NaAlSi_3_O_8(*s*)_ + C_2_H_2_O_4_ + 2H^+^ + 4H_2_O → C_2_O_4_–Al^+^ + 3Si(OH)_4_ + Na^+^
Incongruent weathering	The weathering process that forms clay-sized secondary minerals in addition to dissolved phases as reaction products: 2NaAlSi_3_O_8(*s*)_ + 2H^+^ + 9H_2_O → [Si_2_]Al_2_O_5_(OH)_4(*s*)_ + 4Si(OH)_4_ + 2Na^+^
Weathering congruency	The extent to which mineral weathering results in dissolved vs. solid-phase products. As shown in the two above reactions, weathering congruency can be proxied by the Si/Na and Al/Na ratios of soil porewater.
Weathering intensity	The relative flux of weathering products (e.g., ions of nutrients and ballast elements) per time unit (e.g., per year). Proxies for weathering intensity used in this study include Si flux (*f*_Si_), total cation charge flux, and Sr/Ba ratio.
Weathering steady state	A condition that both weathering intensity and weathering congruency change little during a given period.
Organic matter decomposition rate	The rate by which any organic materials are completely decomposed to inorganic products. The organic matter decomposition rate can be quantified as the integrated fluxes of products such as DIC (CO_3_^2−^, HCO_3_^−^), CO_2_, SO_4_^2−^, NH_4_^+^ (and NO_3_^−^), Cl^−^ and HPO_4_^2−^.
Saturation index (SI)	The saturation status of potentially precipitating secondary phases in an aqueous system, with higher values denoting a greater thermodynamic tendency toward precipitation (see Materials and Methods for more details).
Fluorescence index (FI)	Denoting the origin of DOM, with higher values generally associated with microbially-derived in situ (autochthonous) DOM, and lower values with terrestrially-derived transported (allochthonous) DOM.
Humification index (HIX)	A spectroscopic measure of organic matter transformation by microorganisms, with higher values (stronger humification) corresponding to greater degrees of molecular complexity.
Specific ultraviolet absorbance (SUVA)	A spectroscopic measure of DOM aromaticity, with higher values corresponding to greater DOM aromaticity.

**Fig. 1:**
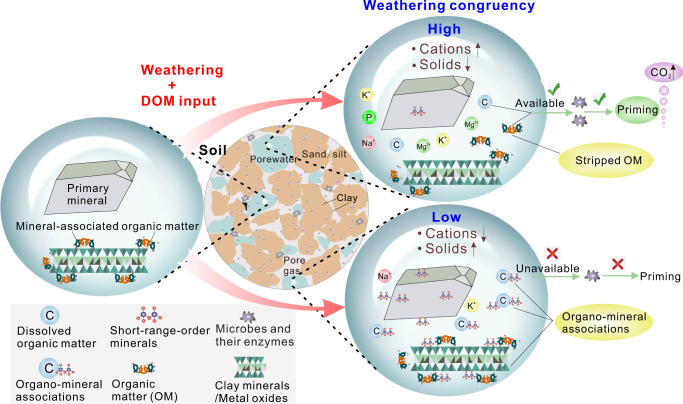
A conceptual model showing the relationship of weathering congruency to the priming effect. Mineral breakdown at high and low weathering congruencies results in different proportions of dissolved vs. solid-phase products (Table 1). High weathering congruency yields more dissolved cations and fewer solids relative to low congruency. Low congruency generates more short-range-order minerals that can bond with and protect organic matter (including dissolved organic matter-DOM) through formation of mineral-organic associations, which are inaccessible to microorganisms and, thus, influence the priming effect. The more limited production of solid phases at high congruency limits bonding and precipitation of dissolved organic matter, thus facilitating the priming of soil organic matter.

Mineral weathering is ongoing ubiquitously in all Earth’s land surface environments. It releases rock-derived nutrients and non-nutrient elements (e.g., K, P, S, Mg, Fe, Ca, Al, and Si and Na) into soil solution and consumes CO_2_ in pore spaces, ranking among the most important processes influencing critical zone evolution, CO_2_ sequestration, and global climate change^26–28^. Mineral weathering supplies microorganisms and plants with rock-derived nutrients, influences the saturation status of secondary minerals with high specific surface area (SSA), and affects the surface characteristics of minerals protecting organic matter (Fig. 1). All these processes may influence microbial activity and/or organic matter bioavailability to microorganisms and, thus, control the primed biodegradation of SOM. However, the mechanisms by which chemical weathering influences soil priming remain unknown.

**Fig. 2:**
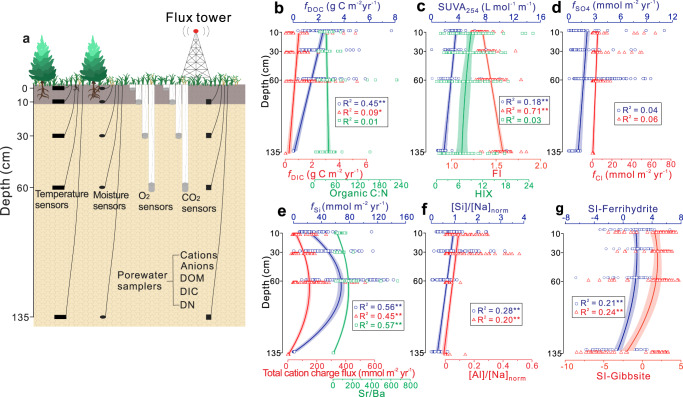
Field monitoring schema and biogeochemical proxy variation as a function of soil depth. **a** Field monitoring of soil temperature, moisture and gas partial pressure, as well as soil porewater sampling. Soil temperature and moisture were monitored at depths of 2, 10, 30, 60, and 135 cm, and soil O_2_ and CO_2_ at 2, 10, 30, and 60 cm. Porewaters were sampled at 10, 30, 60, and 135 cm. Data and depth trends of (**b**) dissolved organic carbon (DOC) flux (*f*_DOC_), dissolved inorganic carbon (DIC) flux (*f*_DIC_) and organic C:N ratio of dissolved organic matter (DOM), (**c**) DOM composition-related parameters including specific ultraviolet absorbance at 254 nm (SUVA_254_), humification index (HIX) and fluorescence index (FI), (**d**) SO_4_^2−^ flux (*f*_SO4_) and Cl^−^ flux (*f*_Cl_), (**e**) proxies for mineral weathering intensity including Si flux (*f*_Si_), total cation charge flux, and Sr/Ba ratio, (**f**) proxies for weathering congruency including [Si]/[Na]_norm_ and [Al]/[Na]_norm_, and (**g**) saturation indices (SI) for secondary minerals ferrihydrite and gibbsite. Proxies for mineral weathering intensity show overall similar depth trends, confirming the predominance of silicate weathering in the soil profile and validating the use of these proxies for analysis of mineral weathering intensity. Weathering has the highest intensity at 60 cm, consistent with the trends of SI of secondary minerals. The data are from all three instrumented pedons. Shaded areas represent 95% confidence intervals. **denotes significant data fit at *p* < 0.01 and *significant data fit at *p* < 0.05.

Deep soil C stocks, i.e., the >50% of organic C that is resident at depths below 1 m, are vulnerable to changes in climate, vegetation successions, and root growth^29,30^. However, biogeochemical processes and their couplings in deep soil are frequently neglected. To advance our understanding of processes operating in the deep soil, we analyzed time series data for porewater chemistry (including organic and inorganic solutes) and soil gas (CO_2_ and O_2_) partial pressures at four depths (i.e., at 10 cm–“topsoil”, 30 cm–“surface soil”, 60 cm–“midsoil”, and 135 cm–“deep soil”) in three instrumented pedons in mixed-conifer forest through long-term, continuous, field-based monitoring, taking advantage of Critical Zone Observatory (Jemez River Basin) infrastructure in New Mexico (Fig. 2a; Supplementary Fig. 1). Our data links mineral weathering with primed SOM decomposition, which – a coupling that plays a key role in controlling soil C dynamics in space and time. The interplay of weathering and priming may represent a link between long-term elemental cycling (=weathering) and fast cycling of carbon and nutrients (=priming).

## Results and discussion

### Vertical soil carbon dynamics and its coupling to mineral weathering

The relationship between humification index (HIX) and DOM biodegradability is often invoked to assert that more condensed aromatic compounds are less biodegradable than compounds with a lower condensation degree^14,31^. Decreasing specific UV absorbance (SUVA) and HIX values with depth point to reduced aromaticity (linked to plant-derived lignin monomers) and molecular complexity with depth (Fig. 2), which is associated with increased prevalence of microbially-sourced organic matter^12,31^. Organic matter in deeper soils typically has a smaller contribution from plants, in particular, from structural compounds such as lignin^31^, which is reflected in changes in the composition and structural properties of DOM compounds with depth (Fig. 2). DOM with lower aromaticity and molecular complexity at depth is more bioavailable to microorganisms, which also explains the increased microbial signal in the fluorescence index (FI) and fluorescence excitation-emission matrices-parallel factor analysis (EEM-PARAFAC) (Fig. 2; Supplementary Fig. 2).

During microbial decomposition of SOM, the thiol (—R—SH) and amino (—R—NH_2_) groups of amino acids from proteins are cleaved to produce HS^−^ and NH_4_^+^, followed by oxidation to SO_4_^2−^ and NO_3_^−^ species^4^. Similar decomposition pathways for other organic compounds (e.g., volatile halogenated organics, chloroacetic acids, and chloromethane) release these and other anions (e.g., Cl^−^) to soil porewater^32–34^. In general, microbial decomposition of organic compounds is an important source of SO_4_^2−^ in soil porewater (in addition to that derived from dissolution of sulfur-bearing minerals). DOC fluxes (*f*_DOC_) and fluxes of SO_4_^2−^ (or Cl^−^; *f*_SO42-_ or *f*_Cl-_) exhibit a close relationship in the midsoil and deep soil, but similar correlations are lacking in the surface soil (Supplementary Figs. 3 and 4). Consequently, the release of Cl^−^ and SO_4_^2−^ below the surface soil, where nutrients, energy and C sources for microorganisms are strongly limited, may be closely linked to the DOM-induced priming effect, although downward transport from the topsoil also represents a source of these anions.

*f*_DOC_ and fluxes of SOM decomposition products such as SO_4_^2−^ and DIC decrease with depth (Fig. 2). These trends are similar to weathering congruency but differ from weathering intensity (Table 1; Fig. 2). Weathering becomes more incongruent deeper in the soil profile (Fig. 2), possibly because, in contrast to Na, Si is precipitated along vertical flow paths—in large (meso- and macro-pores, >50 µm) and micro-pores (>1 µm). Depth trends of weathering intensity are similar to those of the saturation indices (SI) of secondary minerals (Table 1; Fig. 2). These patterns imply that weathering congruency controls SOM decomposition (Fig. 2), establishing a causal linkage between the former and SOM dynamics.

Distributions of *P*_O2_ and *P*_CO2_ with depth in the three instrumented pedons reflect microbial respiration associated with SOM decomposition (Supplementary Fig. 5). *P*_O2_ and *P*_CO2_ generally have opposite trends with depth in each profile. *P*_O2_ decreases and *P*_CO2_ increases to a depth of 30 cm, below which *P*_O2_ increases and *P*_CO2_ decreases (Supplementary Fig. 5). The *P*_CO2_ peak at 30 cm represents the combined effects of microbial and root respiration and limitation of CO_2_ diffusion to the atmosphere. According to vertical trends, *P*_CO2_ is lowest at 135 cm, which is consistent with the *f*_DIC_ result that suggests limited substrate supply and weak SOM decomposition (i.e., CO_2_ and DIC production) at depth (Fig. 2; Supplementary Fig. 5).

### Temporal coupling between organic C dynamics and mineral weathering

**Fig. 3:**
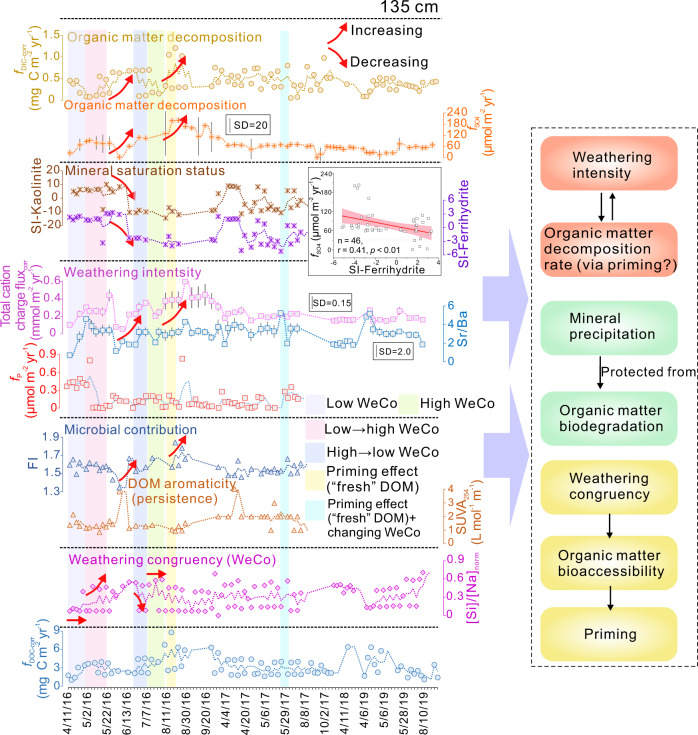
Time series of biogeochemical proxies at 135 cm depth. Proxies for mineral weathering intensity and weathering congruency display a degree of temporal coupling with saturation indices of secondary minerals. These proxies show generally consistent trends with organic matter decomposition rate. In the deep soil, fluxes of SO_4_^2−^ and Cl^−^ are suppressed by precipitation of secondary minerals. Fluorescence index (FI) shows an opposite trend to the parameter that defines dissolved organic matter bioavailability. Fluxes of organic matter decomposition products are similar to the FI time series, suggesting that “primed” microbial activity accelerates organic matter decomposition. The periods with specific biogeochemical characteristics are highlighted with background colors. For example, light purple, light green, pink, and blue bands represent the periods characterized by low, high, low to high, and high to low weathering congruency, respectively, during which specific ultraviolet absorbance at 254 nm (SUVA_254_) and fluorescence index were relatively stable. Small red arrows indicate changing trends (increasing or decreasing) in biogeochemical proxies. The light yellow band defines the period with FI increase, SUVA_254_ decrease and little variation in weathering congruency, which can serve as a control group for evaluating the influence of weathering congruency on the priming effect. Note that the time-series scale is not uniform. Abbreviations: corrected dissolved inorganic carbon (DIC) fluxes—*f*_DIC-corr_, corrected dissolved organic carbon (DOC) fluxes—*f*_DOC-corr_, SO_4_^2−^ fluxes—*f*_SO4_, saturation index—SI, phosphorous fluxes—*f*_P_, normalized Si concentration/Na concentration—[Si]/[Na]_norm_.

In addition to the proxies discussed above, other proxies (e.g., *f*_Si_, *f*_Na_ and total cation charge flux) for mineral weathering intensity generally show consistent temporal trends (Table 1; Fig. 3 and Supplementary Figs. 6–9). These results suggest that silicate mineral dissolution dominates the soil weathering system. The couplings among DOM-related parameters and between weathering and organic C dynamics exist at all soil depths (Fig. 3 and Supplementary Figs. 7–9). Gross primary production (GPP) and net radiation (NETRAD) are two important metrics for plant productivity (Supplementary Fig. 10), which may be the dominant sources of DOM for both aboveground and belowground plant C inputs to the soil. Significant correlations between *f*_DOC-corr_ and HIX were found at 10, 60, and 135 cm (Supplementary Fig. 11), suggesting that pulsed DOM increases are commonly linked to greater inputs of plant inputs characterized by high molecular complexity and aromaticity (i.e., compared to microbial inputs). Considering that HIX is higher in the surface soil, where plant-derived organic matter readily accumulates (Fig. 2), we infer that DOM enrichment in the subsoil is the product of DOM fluxes from the topsoil. In a ^14^C labeling forest mesocosm study^35^, Fröberg et al. demonstrated that a substantial fraction of the subsoil DOM was derived from the topsoil. This supports our argument that the temporal DOM enrichments (Fig. 3 and Supplementary Figs. 6–9) are associated with DOM leaching from the topsoil.

**Fig. 4:**
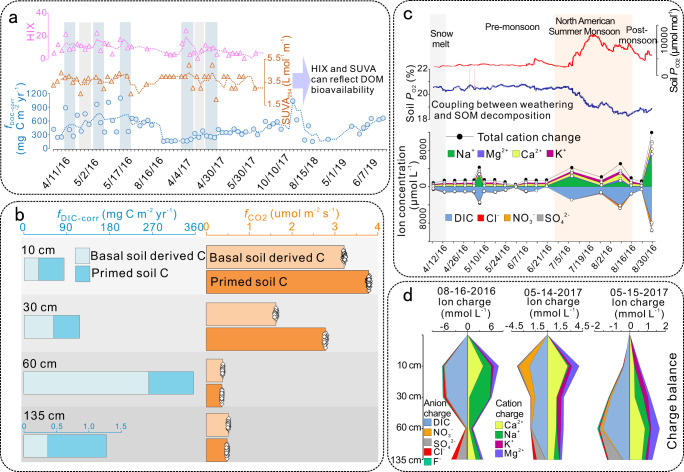
Time series and depth variation. **a** Time series of humification index (HIX), specific ultraviolet absorbance at 254 nm (SUVA_254_) and corrected dissolved organic carbon (DOC) fluxes (*f*_DOC-corr_) in the topsoil. HIX, SUVA_254_ and *f*_DOC-corr_ show generally consistent temporal trends. **b** The priming effect (PE) at four soil depths. The periods for calculation are marked by yellow bands in Fig. 3 and Supplementary Figs. 7–9. The priming effect is calculated using corrected dissolved inorganic carbon (DIC) fluxes (*f*_DIC-corr_) and CO_2_ fluxes (*f*_CO2_) data, with lighter blue or orange representing basal soil-derived C fluxes and darker blue or orange representing “fresh” dissolved organic matter (DOM)-primed C fluxes (i.e., PE _DOM_). The PE _DOM_ in the deep soil is much lower than at shallower depths, yet relative PE _DOM_ is highest in the deep soil. The PE _DOM_ can be used as a “control group” for further evaluation of the influence of mineral weathering on the priming effect. **c**–**d** Temporal and spatial variation in ion charge balance. Time series of CO_2_ partial pressure (*P*_CO2_) and O_2_ partial pressure (*P*_O2_) show inverse temporal trends, suggesting that soil organic matter (SOM) decomposition occurred at the expense of soil O_2_.

Weathering intensity shows generally consistent trends with fluxes of SOM decomposition products (Table 1; Fig. 3 and Supplementary Figs. 7–9). Compared to typical proxies for weathering intensity, phosphorus flux (*f*_P_) is relatively decoupled from SOM decomposition, although *f*_P_ and decomposition rate are often coupled at higher *f*_P_ (Fig. 3 and Supplementary Figs. 7–9). Phosphate in porewater is mainly derived from apatite weathering. Topsoil DOM has the highest HIX and SUVA_254_, and it shows similar trends to *f*_DOC-corr_ (Fig. 4a). This result further confirms that topsoil DOM, which is enriched in aromatic components, was mainly derived from plant litter. This relationship also suggests that HIX and SUVA_254_ can provide robust information on temporal variations in DOM bioavailability^14,31^. Notwithstanding coupling/decoupling between weathering intensity and SOM decomposition, fluxes of SOM decomposition products increase with increased FI and DOM bioavailability (Fig. 3; Supplementary Figs. 7–9). We expect that FI reflects microbial activity boosted by DOM bioavailability because less aromatic DOM is more bioavailable to microorganisms. Drake et al.^36^ suggested that SOM decomposition can be fueled by low-molecular-weight biodegradable DOM, which can be identified by its optical properties. Thus, primed microbial activity, which is a result of low-molecular-weight biodegradable (“fresh”) DOM input, may accelerate SOM decomposition.

### Weathering congruency influences the priming effect

The role of DOM in the priming effect (PE_DOM_) can be assessed by comparing rates of SOM decomposition before and after “fresh” DOM input to a soil during periods of steady state weathering (i.e., only minor changes of weathering congruency and weathering intensity) (Supplementary Fig. 12; Fig. 4b). The periods of PE_DOM_ were selected based on FI increases accompanied by SUVA_254_ drops (yellow bands in Fig. 3 and Supplementary Figs. 7–9; corresponding data shown in Fig. 4b). The PE_DOM_ at 10–60 cm do not vary significantly, especially for primed *f*_DIC-corr_, ranging from 50 to 100 mg C m^−2^ yr^−1^ (Fig. 4b). The PE_DOM_ in the deep soil is much lower compared to that at shallower depths (Fig. 4b). The relative PE_DOM_ in the deep soil, however, is much higher, suggesting that SOM in the deep soil profile responds more sensitively to “fresh” DOM input compared to SOM at shallower depths^24^.

Fluxes of organic decomposition products are suppressed by formation of secondary minerals in the deep soil, whereas this phenomenon was absent at other soil depths (Fig. 3; Supplementary Figs. 7–9). This difference can be ascribed to variation in DOM fluxes, which are low in the deep soil, where DOM precipitates on the highly active surfaces of newly formed secondary clay minerals. At shallower depths, however, DOM fluxes are much larger, and DOM precipitation on newly formed secondary minerals is limited by the availability of the latter. Consequently, the weathering-mediated precipitation of DOM on secondary minerals and with polyvalent metals is crucial for DOM dynamics and organic matter decomposition.

**Fig. 5:**
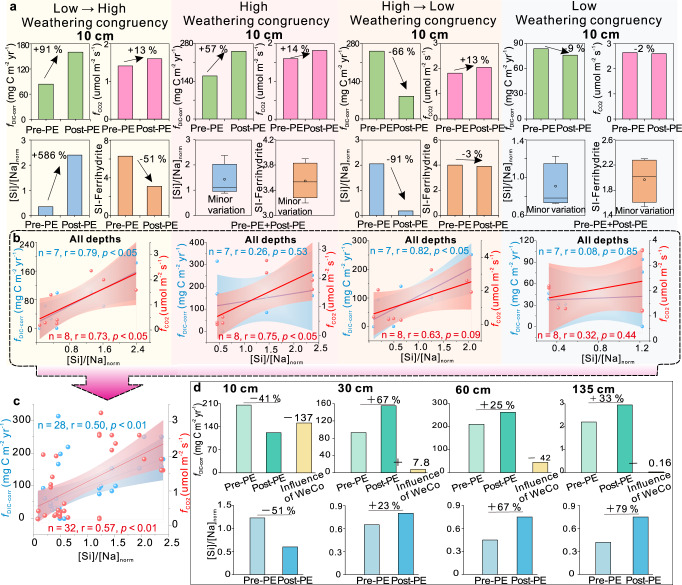
Influence of weathering congruency on organic matter decomposition rate and the priming effect (PE). **a** Indirect linkages between weathering congruency and the priming effect at 10 cm. Normalized Si concentration/Na concentration ([Si]/[Na]_norm_) proxies for weathering congruency, with higher values denoting greater weathering congruency. Four weathering congruency groups are recognized, i.e., low to high weathering congruency group, high weathering congruency group, high to low weathering congruency group, and low weathering congruency group. In most cases, organic matter decomposition rate covaries positively with weathering congruency. **b** Crossplots of organic matter decomposition rate corrected dissolved inorganic carbon (DIC) fluxes (*f*_DIC-corr_) and CO_2_ fluxes (*f*_CO2_) vs. [Si]/[Na]_norm_ for the four weathering congruency groups. **c** Crossplots of organic matter decomposition rate *f*_DIC-corr_ and *f*_CO2_ vs. [Si]/[Na]_norm_ using a compiled dataset from (**b**). **d** Direct influence of weathering congruency on the priming effect. For each depth, the periods for evaluation were selected from Fig. 3 and Supplementary Figs. 7–9 based on similar changes in DOM optical properties to that of “fresh” DOM-induced priming-effect periods without notable change in weathering congruency. *f*_DIC-corr_ generally covaries positively with weathering congruency. For example, in the topsoil, a 51% decrease of weathering congruency leads to a 41% decrease of f_DIC-corr_, resulting in ~137 mg C m^−2^ yr^−1^ decrease of the priming effect.

Weathering intensity and congruency are two parameters characterizing the alteration of primary minerals and controlling the formation of secondary minerals (Table 1; Figs. 1 and 3; Supplementary Figs. 7–9). In the context of weathering congruency, we focus on the [Si]/[Na]_norm_ and SI of secondary minerals. On the one hand, the relationship of weathering congruency to the priming effect can be assessed through its influence on organic decomposition rates (Fig. 5a–c and Supplementary Fig. 13). To exclude the influence of DOM bioavailability and microbial activity on these assessments, we chose periods without notable changes in these parameters (i.e., marked by light purple, light green, pink and blue bands in Fig. 3 and Supplementary Figs. 7–9). Fluxes of organic matter decomposition products increase linearly with weathering congruency (Fig. 5a–c and Supplementary Fig. 13). When weathering congruency increases (the low →high weathering congruency group in Fig. 5a, b), DIC and CO_2_ fluxes also increase. Taking the 10 cm soil depth as an example (Fig. 3), [Si]/[Na]_norm_ increased from 0.35 (4/11/2016) to 2.4 (4/19/2016), resulting in increases of *f*_DIC-corr_ from ~84 to ~160 mg C m^−2^ yr^-1^ and *f*_CO2_ from ~1.4 to ~1.6 μmol m^−2^ s^−1^. In a few cases, *f*_CO2_ and *f*_DIC-corr_ show inconsistent trends, which may be attributed to CO_2_ dissolution in the soil porewater.

The comparison above represents an indirect evaluation of the influence of weathering congruency on the priming effect. On the other hand, we made a direct evaluation of its influence based on the net change in *f*_DIC-corr_ (i.e., net influence = *f*_DIC-corr_ change – PE_DOM_) during periods of strong PE_DOM_ (Fig. 5d). These periods were selected based on similar changes in DOM optical properties to those of “fresh” DOM-induced priming periods during stable weathering congruency. Similarly, the net change in *f*_DIC-corr_ covaries with weathering congruency (Fig. 5d), providing evidence of the magnifying influence of weathering congruency on the priming effect (Fig. 5d). For example, a 41% decrease of weathering congruency in the topsoil is associated with a 51% decrease of *f*_DIC-corr_, resulting in a *ca*. 137 mg C m^−2^ yr^−1^ decrease in the priming effect (Fig. 5d). For the 30 cm soil depth, a 23% increase of weathering congruency is associated with a 67% increase in *f*_DIC-corr_, resulting in a *ca*. 8 mg C m^−2^ yr^−1^ increase in the priming effect (Fig. 5d). Overall, the net influence of weathering congruency decreases with depth. However, considering the low *f*_DIC-corr_ background in the deep soil, the net influence of weathering congruency is greatest in the deep soil and least in the midsoil.

### Mechanisms of weathering effects on priming

Multiple lines of evidence suggest that mineral weathering is linked to soil carbon dynamics via the priming effect: (1) depth profiles show similar trends for weathering congruency, DOC fluxes, and the fluxes of decomposition products (Fig. 2); (2) mineral weathering intensity has a temporal trend similar to that of organic matter decomposition (Fig. 3 and Supplementary Figs. 7–9); (3) organic decomposition rates are temporally coupled with microbial activity and DOM availability, which indicates that “primed” microbial activity accelerates organic matter decomposition; and (4) fluxes of decomposition products linearly correlate with weathering congruency (Fig. 5 and Supplementary Fig. 13).

**Fig. 6:**
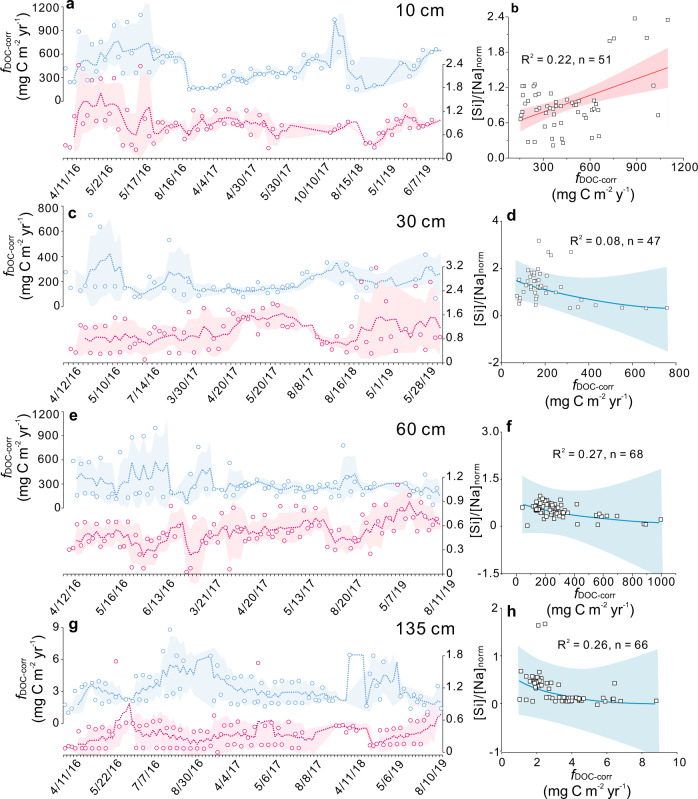
Relationships between dissolved organic carbon (DOC) fluxes and weathering congruency at the four depths. Corrected DOC fluxes (*f*_DOC-corr_) and normalized Si concentration/Na concentration ([Si]/[Na]_norm_) show positive covariation in the topsoil (**a**, **b**) but negative covariation below the topsoil (**c**–**h**). Dashed lines in the time-series plots represent three- or four-point averages. In most cases, three porewater samples were collected from the three pits on each sampling date. Note that the time-series scale is not uniform.

**Fig. 7:**
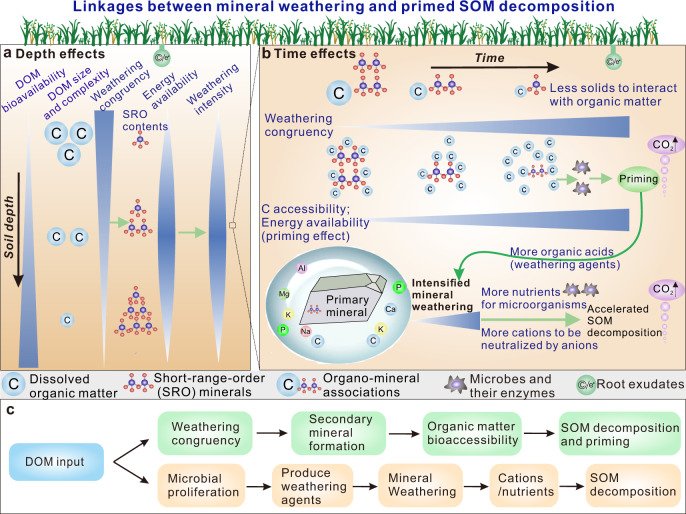
Proposed mechanisms for the priming effect and its linkage to mineral weathering. **a** Depth effect: Mechanisms for linkage between mineral weathering and primed soil organic matter (SOM) decomposition, which is dependent on decreasing dissolved organic matter (DOM) molecular size and complexity and increasing DOM bioavailability with depth. Mineral weathering congruency diminishes with depth because of decreasing DOM molecular size and complexity. The flux and bioavailability of DOM jointly result in a decrease of energy availability with depth, which partly explains vertical changes in weathering intensity (SRO: short-range-order minerals). **b** Time effect: At the temporal scale, weathering congruency generally increases with diminishing DOM molecular size and complexity (i.e., increasing DOM bioavailability). Mineral weathering intensity (fluxes of mineral nutrients) closely controls organic matter decomposition rates, and the priming effect is closely linked to weathering congruency. A likely coupling mechanism is: chemical weathering increases the content of mineral nutrients in porewater for microbial activity, which primes SOM biodegradation. Weathering congruency can exert an important control on the priming effect through influencing the formation of reactive secondary minerals and protecting organo-mineral associations from microbial decomposition (see Fig. 1 for more details). **c** Two possible process sequences of DOM input to soil, which are summarized from Critical Zone observations.

One of the most striking findings is that DOM can influence the type of chemical weathering, i.e., congruent weathering vs. incongruent weathering. Weathering congruency increases with organic ligand concentrations in the topsoil (Fig. 6), reflecting decreasing formation of secondary clays (Fig. 6), but it decreases with *f*_DOC-corr_ below the topsoil. Because DOM is the driver of priming, DOM links weathering with priming. These responses of weathering congruency to *f*_DOC-corr_ may be ascribed to the complexity, aromaticity and flux of DOM with depth (Fig. 2): DOM in the topsoil has complex structure and high aromaticity, which favor more congruent mineral weathering (i.e., releasing more cations and forming fewer nanosized secondary mineral phases on organo-mineral surfaces). These processes create a microenvironment that is less protective of mineral-associated organic matter and, therefore, more favorable for the priming effect (Fig. 1). DOM at greater soil depths has a less complex structure and lower aromaticity (Fig. 7), likely contributing to incongruent weathering (i.e., forming more nanosized secondary mineral phases on mineral surfaces that protect organic matter from decomposition).

Weathering congruency can influence mineral surface properties and precipitation of organics on secondary minerals, thereby influencing microbial access to organic matter and, hence, the priming effect (Figs. 1 and 7). Weathering is coupled to organic matter decomposition rate through changes in the availability of nutrients, especially phosphorus (Fig. 7), which is an important factor for microbial growth and metabolism^37^. Soil microorganisms invest energy and nutrients to produce extracellular enzymes (exoenzymes) that decompose complex organic compounds^37,38^. Thus, mineral weathering can be directly linked to the priming effect and soil carbon dynamics through its influence on nutrient supply (Fig. 7). A consequence of greater rock-derived nutrient inputs is primed respiration of heterotrophs and organic matter decomposition (Fig. 7), which jointly maintain cation-charge balance (Fig. 4c, d). This mechanism is supported by positive covariation of soil *P*_CO2_ with total cation charge—both soil *P*_CO2_ and total cation charge are notably higher during/after the summer monsoon compared to pre-monsoon (Fig. 4c). *P*_CO2_ and total cation charge also generally covary with *P*_O2_, indicating that O_2_ is the main electron acceptor during organic matter oxidation. Another mechanism for coupling weathering with organic matter decomposition via priming may be the retardation of weathering owing to insufficient organic C for microorganisms producing weathering agents (e.g., organic acids^39^) (Fig. 7).

### Implications for terrestrial C cycling

Our results have important implications for global carbon cycling. First, biogeochemical couplings at a range of temporal and spatial scales (Fig. 7) reflect the influence of weathering on SOM priming. Many previous studies neglected the influence of mineral weathering and depth-dependent soil processes on priming. Karhu et al.^17^ suggested that deep soil is more susceptible to priming due to increases in C and N availability. Because absolute priming is greatest in the topsoil, its influence on the deep soil is frequently neglected, even though relative priming can be greater at depth. Although Garcia Arredondo et al.^30^ found that plant-driven weathering influenced soil C dynamics, the results of our study based on continuous field monitoring of soil gas and porewater fluxes in instrumented pedons establish an unambiguous link between mineral weathering and the priming effect. The data confirm that the priming effect is common in plant-soil systems and not an artifact of incubation studies^20^.

Other implications are related to the importance of deep soils in the global C cycle. More than half of total organic C stocks reside at soil depths of >1 m. Our study demonstrates that, despite limited fluxes of water, nutrients, and energy, the relative priming effect is most intense in the deep soil (Fig. 4) and it is intimately connected with mineral weathering. These findings have not been reported previously because (1) direct, field-based monitoring of multiple soil processes (especially organic matter transformations and DOM fluxes) at a range of soil depths has rarely been achieved, and (2) the soil below 30–50 cm is neglected in most studies. Thus, our study represents a unique contribution to soil science based on a large, integrated dataset from a long-term field experiment, offering significant advantages compared to largely lab-based priming studies. Carbon storage in the deep soil is highly sensitive^24^ to porewater fluxes containing fresh organics. Semi-arid and sub-humid ecosystems are viewed as a net global terrestrial CO_2_ sink, with montane forests sequestering the majority of the C, thus playing a key role in modulating atmospheric CO_2_^40,41^. Consequently, disruption of the C balance in the deep soil runs a significant risk of CO_2_ release and exacerbation of climate change in the future. Semi-arid and sub-humid soils have the potential to shift from a net CO_2_ sink to a net CO_2_ source if, for example, increased rainfall induced higher soil-water availability and DOM fluxes at depth.

The study site is subject to seasonal dry/wet cycles and is well drained, making it suitable for investigation of the SOM priming effect by DOM fluxes. Priming is ubiquitous even in water-logged conditions as in paddy soils^20,42^^,^. It is induced primarily by labile C input rather than soil water flux, and thus occurs even in water-logged conditions. We suggest that the linkages between weathering and priming (Figs. 1 and 7) are transferable to many soils having a broad range of moisture conditions. Weathering produces not only rock-derived nutrients but also nano- to clay-sized secondary minerals. Mineral weathering and organic matter dynamics are two critical processes that regulate soil formation, nutrient cycling, CO_2_ emissions, and climate change, linking C fluxes between the Earth’s surface and crustal reservoirs^43,44^. The soil components controlling these processes—minerals and SOM—are two most abundant solid materials in pedons globally. Thus, we anticipate that similar causal relationships between weathering and priming are common in most soils. Under water-limited conditions as in the JRB-CZO, both organic matter decomposition and mineral weathering are relatively slow. With greater water availability as in wetter soils, mineral weathering, the priming effect and their interactions can become intensified. Interactions between weathering and priming, which have not been adequately investigated to date, may represent fundamental links between long-term elemental cycling (=weathering) and the rapid turnover of soil C and nutrients (=priming) on Earth.

## Methods

### Study site

The fieldwork was conducted in the Jemez River Basin Critical Zone Observatory (JRB-CZO) within the Valles Caldera National Preserve in the Jemez Mountains in north-central New Mexico^45^ (Supplementary Fig. 1). The JRB has an elevation of 1700-3432 m a.s.l., and at its upper elevations it contains montane mixed-conifer forests that are common high-elevation ecosystems throughout the western United States. The study site is a mature forested low-gradient hillslope that drains into a first-order channeled basin^45^. This study site was established in the fall of 2015. It contains an instrumented flux tower (Ameriflux) and three instrumented pedons located ~20 m from the flux tower (Supplementary Fig. 1). Canopy heights range from 10 to 31 m around the flux tower. The three pedons were excavated and instrumented in August 2015 (Supplementary Fig. 1). The parent material of the soils is rhyolitic tuff.

The Jemez Mountains are located in the transition zone between southwestern US and the snow-dominated Rocky Mountains^46,47^. Thus, the Jemez Mountains have a bimodal pattern of annual precipitation, with approximately half of annual precipitation falling as winter snow, and the other half as rain^45^. This region has a typical semiarid and montane climate with annual precipitation ranging from 600 to 1021 mm and mean annual precipitation of 777 mm. Air temperature at the study site ranges from –15 °C in the winter to 25 °C in the summer, with average winter and summer temperatures of ~–1 °C and 11 °C.

### Meteorological measurements and monitoring of soil microclimate

Flux tower-based meteorological measurements of above-ground ecosystem-atmosphere exchanges of water and energy fluxes have been conducted since January 2016 in the ZOB (Ameriflux Site ID: US-Vcs). Here, we focus only on data of precipitation, net radiation (NETRAD), and gross primary production (GPP, which is equivalent to apparent photosynthesis, i.e., net ecosystem exchange minus ecosystem respiration). Meteorological measurements enable correlation between above- and below-ground processes.

Co-located sensor and sampler instruments were installed in the instrumented pedons (Fig. 2). Soil moisture and temperature were quasi-continuously monitored at depths of 2, 10, 30, 60, and 135 cm within the three instrumented soil pits (located ~20 m from the AmeriFlux tower) from October 2015 to October 2017. These data were measured at 15 min intervals and were stored in CR1000 dataloggers (Campbell Scientific, Logan, Utah, USA). Soil moisture and temperature were measured using Decagon 5TE sensors (Decagon Devices Inc., Pullman, Washington, US).

### Measurement of soil O_2_ and CO_2_ partial pressures

Partial pressures of soil O_2_ and CO_2_ were continuously measured at 2, 10, 30, and 60 cm within the three pedons using Apogee SO-110 (galvanic cell) and solid-state infrared gas sensors GMP-222 (Vaisala Inc., Helsinki, Finland), respectively. Partial pressures of soil CO_2_ (*P*_CO2_) recorded by the sensor were corrected for variations in pressure and temperature^48^ as follows:

1 $${C}_{c}={C}_{m}-{C}_{P}-{C}_{T}$$

where *C* represents the partial pressure of CO_2_, and the subscripts c, m, P, and T represent corrected, measured, pressure correction, and temperature correction, respectively. The atmospheric pressure-corrected *P*_CO2_, C_p_, was calculated as:

2 $${C}_{P}={K}_{P}\left[\frac{P-101.3}{101.3}\right]$$

where P (kPa) is the atmospheric pressure recorded by flux tower and K_P_ (ppm) was calculated as:

3 $${K}_{P}=1.38\times {C}_{m}$$

The temperature-corrected *P*_CO2_, *C*_*T*_, was calculated as:

4 $${C}_{T}=14000 \, ({K}_{T}-{K}_{T}^{2})\left[\frac{25\,-\,{T}_{c}}{25}\right]$$

where Tc is the soil temperature (°C) and K_T_ (ppm) was calculated as:

5 $${K}_{T}={{{{{{\rm{A}}}}}}}_{0}+{{{{{\rm{A}}}}}}_{1}\times {C}_{m}+{{{{{\rm{A}}}}}}_{2}\times {{C}_{m}}^{2}+{{{{{\rm{A}}}}}}_{3}\times {{C}_{m}}^{3}$$

with A_0_ = 3 × 10^−3^ ppm, A_1_ = 1.2 × 10^−5^, A_2_ = –1.25 × 10^−9^, A_3_ = 6 × 10^−14^.

Similarly, partial pressures of soil O_2_ (*P*_O2_) were also corrected for variations in pressure and temperature.

### Calculation of soil CO_2_ efflux

We used the gradient method, which is based on the gradient of soil CO_2_ concentration and effective CO_2_ diffusivity of the soil to calculate soil CO_2_ efflux^48,49^. The CO_2_ flux (F) at a depth of *z* can be calculated using Fick’s first law of diffusion:

6 $${F(z)}={-}{{{{{{\rm{D}}}}}}}_{s}\frac{{dC}}{{dz}}$$

where D_s_ is the effective CO_2_ diffusivity within the soil (m^2^ s^−1^), $\frac{d{{{{{\rm{C}}}}}}}{d{{{{{\rm{z}}}}}}}$ the vertical CO_2_ gradient, and the negative sign indicates that the efflux is decreasing upwards. C (μmol m^−3^) here can be converted from corrected *P*_CO2_ (μmol mol^−1^) by:

7 $${C}=\frac{{C}_{c}P}{{{{{{\rm{R}}}}}}T}$$

where *R* is the universal constant (8.314 J mol^−1^ K^−1^), T the soil temperature (K), and P the air pressure (Pa). D_s_ can be calculated as:

8 $${D}_{s}=\xi {{{{{{\rm{D}}}}}}}_{a}$$

9 $${{{{{{\rm{D}}}}}}}_{a}={{{{{\rm{D}}}}}}_{a0}\left(\frac{T}{293.2}\right)^{1.75}\left(\frac{101.3}{P}\right)$$

where D_a_ is the CO_2_ diffusion coefficient in the air, D_a0_ a reference constant (1.47 × 10^−5^ m^2^ s^−1^) at 101.3 kPa and 293.2 K, and ξ the gas tortuosity factor. Several models have been proposed for computing ξ, and we chose the model by Moldrup et al.^50^ (2000) for our samples because it often produces results in good agreement with chamber-based estimates:

10 $${{{{{\rm{\xi }}}}}}=\frac{{({{{{{\rm{\varphi }}}}}}-{{{{{\rm{\theta }}}}}})}^{2.5}}{{{{{{\rm{\varphi }}}}}}}$$

where φ represents the porosity, and θ the volumetric water content. The difference between these two parameters is equal to air-filled porosity (ε):

11 $${{{\rm{\varphi }}}}={{{{{\rm{\varepsilon }}}}}}+{{{{{\rm{\theta }}}}}}=1-\frac{{\rho }_{s}}{{\rho }_{m}}$$

where *ρ*_*s*_ is the bulk density of the analyzed soil, and *ρ*_*m*_ is the particle density of the mineral soil, with an assumed value of 2.65 g cm^−3^. Combining these equations, we can calculate CO_2_ flux (*f*_CO2_) at depth *z* (*F(z)*).

### Calculation of water flux

Soil water flux at each soil depth can be directly calculated from passive capillary sampler (PCAP) datasets. The PCAPs, which are passively collecting water flux data at all times, are excellent for calculation of water flux because of the nature of the hanging water column. From those PCAPs, we calculated the volume of water passing through the soil profile per unit area to get water flux data. Specifically, water flux (*f*_H2O_) was calculated by dividing the volume collected in the PCAP carboy by the cross-sectional area of the PCAP plate (7.8  × 10^−2^  m^2^), which gives the volume flux of water for the period of collection (T) that contributes to recharge:

12 $${f}_{{{\rm{H2O}}}}=V/(T\times 7.8\,\times {10}^{-2}{{{{{{\rm{m}}}}}}}^{2})$$

Solute flux (e.g., *f*_SO4_ and *f*_DIC_) was calculated by multiplying solute concentration (e.g., [SO_4_^2−^] and [DIC]) by water flux. Because water flux was calculated using integrated data for a whole year, we generated only a single water flux datum for a specific depth. However, water flux should fluctuate through each year due to temporal variability in rainfall and soil moisture content. Thus, we also multiplied solute flux by soil moisture to get moisture-corrected solute flux data (e.g., *f*_SO4-corr_ and *f*_DIC-corr_).

### Collection of soil-porewater and precipitation samples

Soil porewaters were collected in situ weekly or biweekly throughout the rainy season (March to October) during 2016 and 2017 and for more limited intervals during 2018 and 2019. They were collected with two types of soil porewater samplers, i.e., Prenart Super Quartz tension lysimeters (2 μm pore size, Prenart Equipment ApS, Denmark) and fiberglass wick-based passive capillary wick samplers (PCAPs^51^). These samplers have proven effective for soil porewater sampling and suitable for analysis of DOM^51^. The Prenart Super Quartz tension lysimeters were installed at depths of 10, 30, 60, and 135 cm, and the PCAPs were installed at depths of 10, 30, 60 cm in each soil pit. These installations provided vertically and temporally resolved porewater samples. For Prenart Super Quartz tension lysimeters, a vacuum pump applied a constant suction of 40 kPa to collect soil solution samples into high-density polyethylene (HDPE) bottles. PCAPs consist of a fiber-glass wick covered with a HDPE plate. PCAPs were used to collect soil solutions as a function of depth in each pit under a constant negative pressure head of ~30 cm (~2.9 kPa). Precipitation samples were collected on 15 March 2015 from four snowpacks (with samples taken at three depths within each snowpack) near the flux tower and three soil pits.

### Soil-porewater and precipitation chemistry analyses

All soil-porewater and precipitation samples were kept frozen and sent overnight to the University of Arizona Laboratory for Emerging Contaminants to be filtered within 48 h of collection using 0.45 μm nylon filters. Sample aliquots for DOM and DIC were filtered through combusted 0.7 μm glass-fiber filters. Concentrations of DOC ([DOC]) and DIC ([DIC]) were determined by infrared detection of CO_2_ after catalysis at 720 °C (DOC) or aridification (DIC) using a Shimadzu TOC-VCSH carbon analyzer (Columbia, MD). DIC, an important parameter in this study, was defined as the sum of H_2_CO_3_^aq^ (carbonic acid), [HCO_3_^−^], [CO_3_^2−^], and soil CO_2_, which would react with porewater to form DIC:

13 $${{{{{{{\rm{CO}}}}}}}_{2}}^{{{{{{\rm{gas}}}}}}}+{{{{{{\rm{H}}}}}}}_{2}{{{{{\rm{O}}}}}} \, \rightleftharpoons \, {{{{{{\rm{H}}}}}}}_{2}{{{{{{{\rm{CO}}}}}}}_{3}}^{{{{{{\rm{aq}}}}}}} \, \rightleftharpoons \, {{{{{{\rm{H}}}}}}}^{+}+{{{{{{{\rm{HCO}}}}}}}_{3}}^{-} \, \rightleftharpoons \, 2{{{{{{\rm{H}}}}}}}^{+} \,+\, {{{{{{{\rm{CO}}}}}}}_{3}}^{2-}$$

where H_2_CO_3_^aq^ represents the hydrated form of dissolved CO_2_.

The speciation of Eq. (13) is mediated by soil porewater pH: for a soil pH < 6.4, H_2_CO_3_* dominates DIC, with HCO_3_^−^ dominating at 6.4 < pH < 10.3^52^. Per Henry’s law, the amount of dissolved CO_2_ in water is proportional to the soil *P*_CO2_:

14 $${{P}_{{{\rm{CO2}}}}}^{{{{{{\rm{gas}}}}}}} \, \rightleftharpoons \, {K}_{H}\times [{{{{\rm{CO}}}}_{2}}^{{{{{{\rm{aq}}}}}}}]$$

15 $$[{{{{{{{\rm{CO}}}}}}}_{2}}^{{{{{{\rm{aq}}}}}}}]+{{{{{{\rm{H}}}}}}}_{2}{{{{{\rm{O}}}}}} \, \rightleftharpoons \, {{{{{{\rm{H}}}}}}}_{2}{{{{{{{\rm{CO}}}}}}}_{3}}^{{{{{{\rm{aq}}}}}}}$$

where *K*_*H*_ represents Henry’s law constant for CO_2_ (34.06 mol m^−3^ atm^−1^) at 25 °C. Henry’s law implies that DIC can reflect the *P*_CO2_ in soil pore spaces and thus the intensity of microbial decomposition of SOM to DOM via soil heterotrophic respiration.

 Concentration of dissolved nitrogen ([DN]) was measured by nitrate colorimetric analysis after high-temperature combustion of all N species to nitrate. We assume that inorganic N is mainly present in the form of NO_3_^−^, and thus the concentration of dissolved organic nitrogen ([DON]) can be calculated as the difference between [DN] and the concentration of NO_3_^–^N. Concentrations of anions (e.g., Cl^−^, NO_3_^−^, and SO_4_^2−^; abbreviated as [Cl^−^], [NO_3_^−^], and [SO_4_^2−^], respectively) were analyzed by ion chromatography using Dionex ICS-1000 equipped with an AS22 Analytical Column, Sunnyvale, CA. Concentrations of cations and silicon were determined by ICP-MS (ELAN DRC-II, Shelton CT).

Soil porewater inorganic chemistry data were used to perform aqueous geochemical modeling with MINTEQ3.1. The modeling involves determination of aqueous phase speciation and calculation of SI values of pore waters with respect to various potentially precipitating secondary phases. The SI was calculated as:

16 $$SI={{{\rm{log}}}}\,\varOmega={{{\rm{log}}}} \, IAP \, {-} \, {{{\rm{log}}}}\,{K}_{sp}$$

where *Ω* is the relative saturation, *IAP* is the ion activity product, and *K*_*sp*_ is the solubility product constant at a given temperature. Positive SI specifies supersaturation and precipitation of the solid, and negative SI specifies undersaturation and dissolution. DOC concentration and site density (2.4 × 10^−6^ mol of sites per mg DOC) were used as input parameters to account for metal-ligand complex formation.

### Plotting of time series

Time series of mineralogical and organic and inorganic geochemical parameters were plotted for four depths within the instrumented pedons (10, 30, 60 and 135 cm) using filtered data. For each sampling date, data for the three pedons are spread out slightly on the temporal axis for display purposes—for example, data for 4/19/2016 are plotted as 4/18/2016 for pit 1, 4/19/2016 for pit 2, and 4/20/2016 for pit 3. Because the distribution of sampling dates is uneven, we used a ‘homogenization’ function in Excel to make the plots more uniform, rendering the dates on the *x*-axis unevenly distributed but improving the visual aspect of the plots. These graphical transformations did not alter any data in the porewater geochemical dataset. In a few cases (e.g., time series of total cation charge, Sr/Ba and *f*_SO4_), mean values were used to better compare information conveyed in different types of plots.

### Calculation of weathering congruency and priming effect

Products of congruent weathering are only dissolved species, whereas products of incongruent weathering also contain solid phases (Table 1). The relative contribution of weathering congruency vs. incongruency can be measured by normalizing element release to that of dissolved sodium^53^, which is considered a “conservative” tracer of mineral dissolution, because it does not re-incorporate into secondary solid structures, and it is a weak competitor for cation exchange sites. Here we use the ratio of {[Si]_solution_/[Na]_solution_}/{[Si]_solid_/[Na]_solid_} (simplified as [Si]/[Na]_norm_) to provide a normalized measure of weathering stoichiometry (weathering congruency). The priming effect caused by fresh DOM input can be quasi-quantitatively estimated by comparing *f*_DIC-corr_ and *f*_CO2_ before and after the introduction of “fresh” DOM during the period with very minor change in weathering congruency (Supplementary Fig. 12):

17 $${Absolute}\,{P}{{E}}_{{DOM}}={Respons}{{e}}_{^{{\prime\prime}} {fresh}^{\prime\prime}} {DOM} \, {-} \, {Respons}{{e}}_{^{{\prime\prime}} {control}^{\prime\prime} }$$

18 $${Relative}\,{P}{{E}}_{{DOM}} (\%)=\frac{{Response}_{^{\prime\prime} {fresh}^{\prime\prime}} {DOM} -{Response}_{^{\prime\prime} {control}^{\prime\prime}} }{{Response}_{^{\prime\prime} {control}^{\prime\prime}} }\times 100$$

where Response_“fresh” DOM_ and Response_“control”_ represent *f*_DIC-corr_ (mg C m^−2^ yr^−1^) and *f*_CO2_ (μmol m^−2^ s^−1^) after and before the input of more bioavailable (“fresh”) DOM, respectively. The criteria for selection of the period of the priming-effect calculation is a notable increase in FI (microbial activity) accompanied by diminishing SUVA_254_ (increasing bioavailability) with minor variation in weathering congruency, as this kind of period may represent a “fresh” DOM-induced priming process. Thus, *f*_DIC-corr_ and *f*_CO2_ at the beginning of this period can be viewed as a control group for the calculation of the priming effect at different depths.

After calculating the priming effect at different depths, we quantify the influence of mineral weathering on the priming effect. For a specific soil depth, we view the period with relatively low [Si]/[Na]_norm_ values as a “control group” with low weathering congruency. It is worth noting that the priming effect in the control group was not unimpacted by chemical weathering but it had almost lowest weathering congruency. Another precondition for the selection of the “control group” is that corresponding FI values should be relatively low, that is, microbial activity has not been primed by bioavailable DOM. The influence of chemical weathering (congruency) on the priming effect can be assessed through comparing SOM decomposition rate (including *f*_DIC-corr_ and *f*_CO2_) in “experimental” groups with varying degrees of weathering congruency (i.e., low→high, high→low and high weathering congruency) and control group with low weathering congruency. In particular, we focus on the response of SOM decomposition rate to weathering congruency. In order to exclude the influence of variation of DOM bioavailability on our assessment, we chose the periods with only minor change in SUVA_254_ (DOM bioavailability) and FI (microbial activity) values for each “experimental group”.

### Spectroscopic analyses and PARAFAC modeling

Molecular characterization of DOM was conducted using UV-Vis spectroscopy with Shimadzu Scientific Instruments UV-2501PC (Columbia, MD, United States) and fluorescence excitation-emission matrices (EEMs) (FluoroMax-4 equipped with a 150 W Xe-arc lamp source, Horiba Jobin Yvon, Irvine, CA, United States). Samples for UV-Vis spectroscopy analysis were placed in 1 cm path-length quartz cuvettes. The SUVAs at 254 nm (SUVA_254_) and at 280 nm (SUVA_280_) were calculated by dividing the UV absorbances measured at 254 nm and 280 nm with the cell path length and DOC concentration. All samples had absorbance reading <0.4 at 254 nm. Because iron also absorbs light at 254 nm, relatively high concentrations of Fe (e.g., >500 μg L^−1^) can cause incorrect SUVA values^10,54^. However, we confirmed that the vast majority of data (>98%) had Fe concentrations much lower than 500 μg L^−1^.

EEMs for DOC samples were used to calculate informative optical indices for DOC quality. EEMs were collected at a 5 nm step size over an excitation (Ex) range of 200–450 nm and an emission (Em) range of 250–650 nm. Spectra were collected with Em and Ex slits at 2 and 5 nm band widths, respectively, with an integration time of 100 nm. Ultrapure water blank EEMs were subtracted, and fluorescence intensities were normalized to the area under the water Raman peak. An inner-filter correction was performed according to the corresponding UV-Vis scans^55^.

Fluorescence index (FI) was calculated as the ratio of the emission intensity at 450 nm and 500 nm using corrected EEMs^56^:

19 $${{{{{\rm{FI}}}}}}_{{{{{{\rm{Ex}}}}}}370}=\frac{{I}_{450}}{{I}_{500}}$$

where Ex is the excitation wavelength (nm) and I the emission intensity at each wavelength. Fluorescence spectroscopy was also used to determine the extent of humification by quantifying the extent of shifting of the emission spectra toward longer wavelengths with increasing humification^55^. Humification index (HIX) was estimated by the ratio of the area of emission spectra (435–480 nm) to that of emission spectra (300–345 nm) using an EX wavelength at 255 nm^55^:

20 $${{{{{\rm{HIX}}}}}}_{{{{{{\rm{Ex}}}}}}255}=\frac{\sum ({I}_{435\to 480})}{\sum ({I}_{300\to 345})}$$

PARAFAC modeling can take overlapping fluorescence spectra and decompose the data into score and loading vectors. An original PARAFAC model was derived from hundreds of fully corrected EEMs from soil porewater, stream water, and precipitation samples from the JRB CZO^57^. PARAFAC modeling of fluorescence EEMs was conducted using the DOMFluor toolTable in Matlab following the protocol and quality control procedure described in Stedmon and Markager^58^.

### Solid phase analyses

Soils and soil porewater samples were taken at depths of 5–10 cm (topsoil), 20–30 cm (surface soil) 40–60 cm (midsoil), and 120–140 cm (deep soil) (Supplementary Table 1). The soil samples were air-dried and sieved to isolate the <2 mm fine earth fraction prior to characterization. Soil pH was measured at mass to volume ratios of 1:1 (soil:water) solution. Particle-size distribution was analyzed by laser diffraction using a Mastersizer 3000 laser particle-size analyzer following pretreatment with 30% H_2_O_2_ to remove organic matter and dispersion with 5% sodium hexametaphosphate. Total C and N measurements were made using a Finnigan Delta Plux XL coupled to an elemental analyzer. Total C and N were assumed to equal to organic C and N because of no evidence for carbonates. Selective extraction was performed on the fine-earth soil fraction using sodium pyrophosphate (PP), acid ammonium oxalate (OX) and dithionite-citrate to determine the contents of Fe and Al phases. The Brunauer–Emmett–Teller SSA of soil fine earth fraction was determined using isotherm and N_2_ gas adsorption by Micromeritics 160202. The samples were degassed prior to measurement by heating at 130 °C and purged by N_2_ gas for ~3 h.

## Supplementary information

Supplementary Information

Description of Additional Supplementary Files

Supplementary Data 1

Supplementary Data 2

Supplementary Data 3

Supplementary Data 4

Supplementary Data 5

Supplementary Data 6

Supplementary Data 7

Supplementary Data 8

Supplementary Data 9

Supplementary Data 10

## Data Availability

The authors declare that data supporting the findings of this study are available within the paper and its supplementary information files.
