# Supplementary material for: Mineral weathering is linked to microbial priming in the critical zone
Source: Nat Commun. 2023 Jan 20;14:345. doi: 10.1038/s41467-022-35671-x (PMC9860040; doi:10.1038/s41467-022-35671-x)
Supplement: Supplementary file 1 — Supplementary Information [file 41467_2022_35671_MOESM1_ESM.pdf]

Supplementary Figures 1–13, Supplementary Table 1, Supplementary Notes 1–2

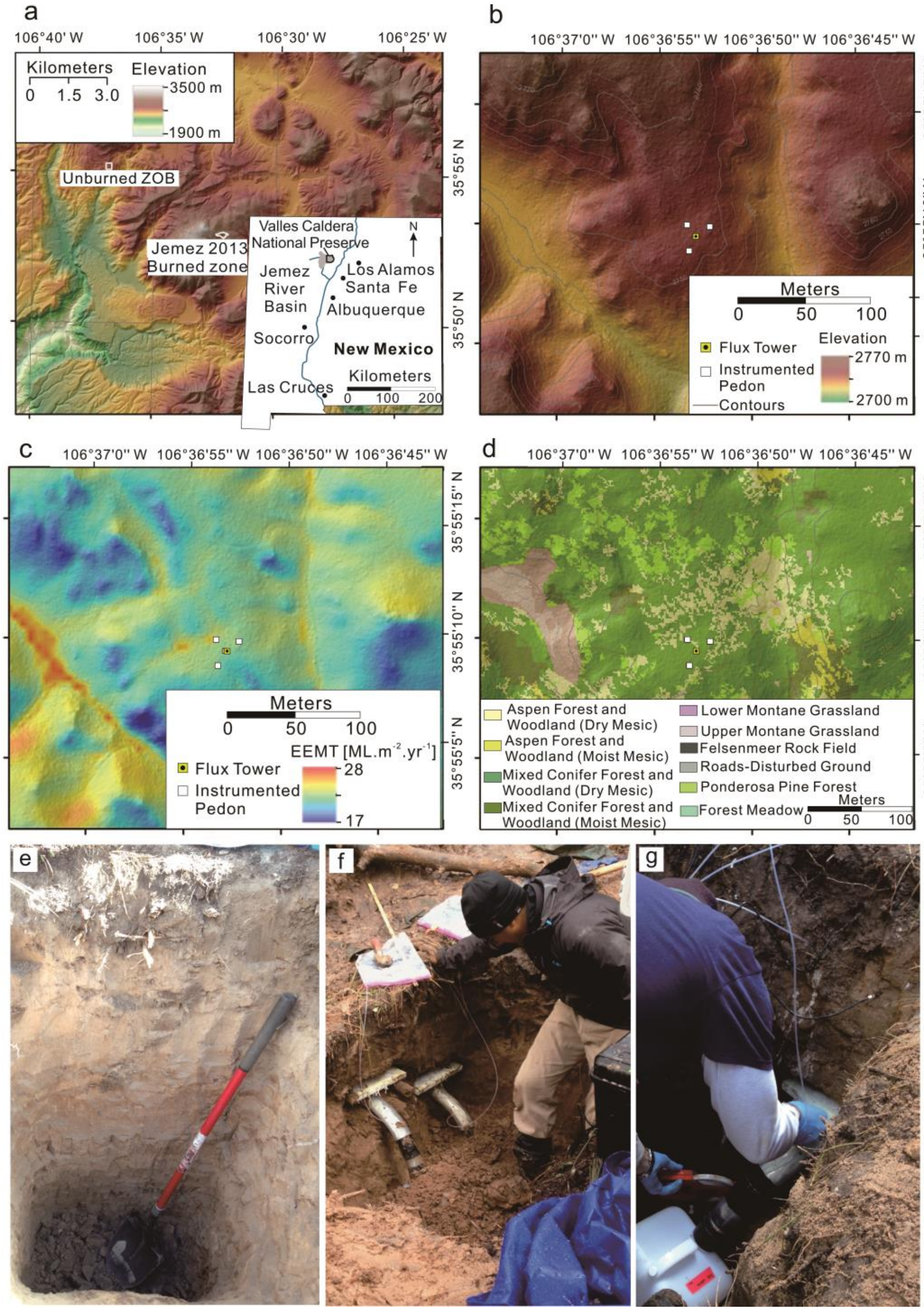

Supplementary Fig. 1 Location map and field monitoring and sampling schematic and photos. (a) Location and topography of the Sulfur Ridge mixed conifer site (red rectangle) on San Antonio Mountain in the Valles Caldera National Preserve (VCNP), Jemez River Basin (JRB), New Mexico. (b) Light detection and ranging (LiDAR)-derived digital elevation map (DEM), (c) environmental energy and mass transfer (EEMT) annual flux calculations, and (d) vegetation classes of study site. Also shown in (b)-(d) are the locations of the three soil pedons, each ~20 m from and surrounding the flux tower. (e-g) Photos showing soil pits and installation of sensor and porewater samplers.

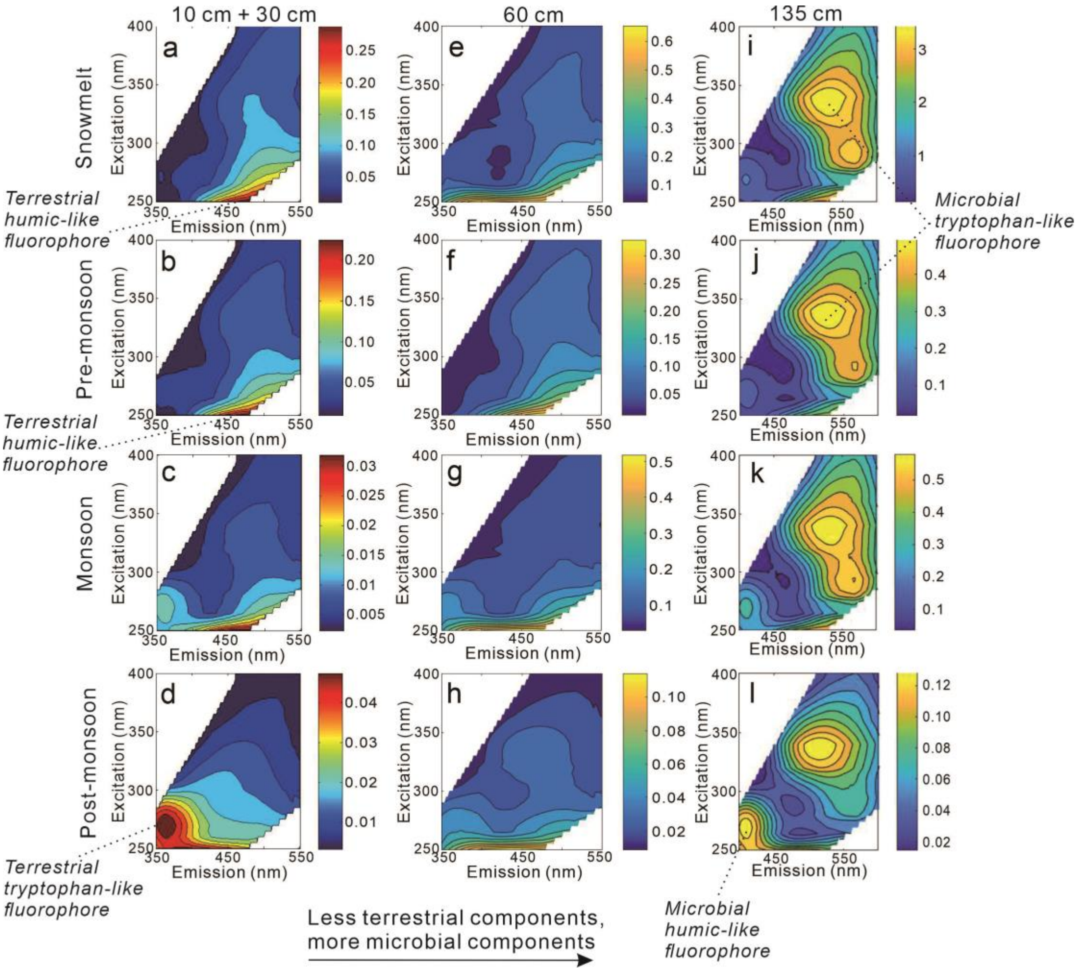

Supplementary Fig. 2 The excitation-emission matrixes for soil porewater dissolved organic matter. These soil porewaters were sampled from different seasons (i.e., snowmelt, pre-monsoon, monsoon, and post-monsoon) in (a-d) 10 cm + 30 cm, (e-h) midsoil (60 cm), (i-l) deep soil (135 cm). The excitation-emission matrix-parallel factor analysis components identified in this study show obvious variations with time and soil depth. Porewater samples from a specific soil horizon have generally similar excitation-emission matrix spectra.

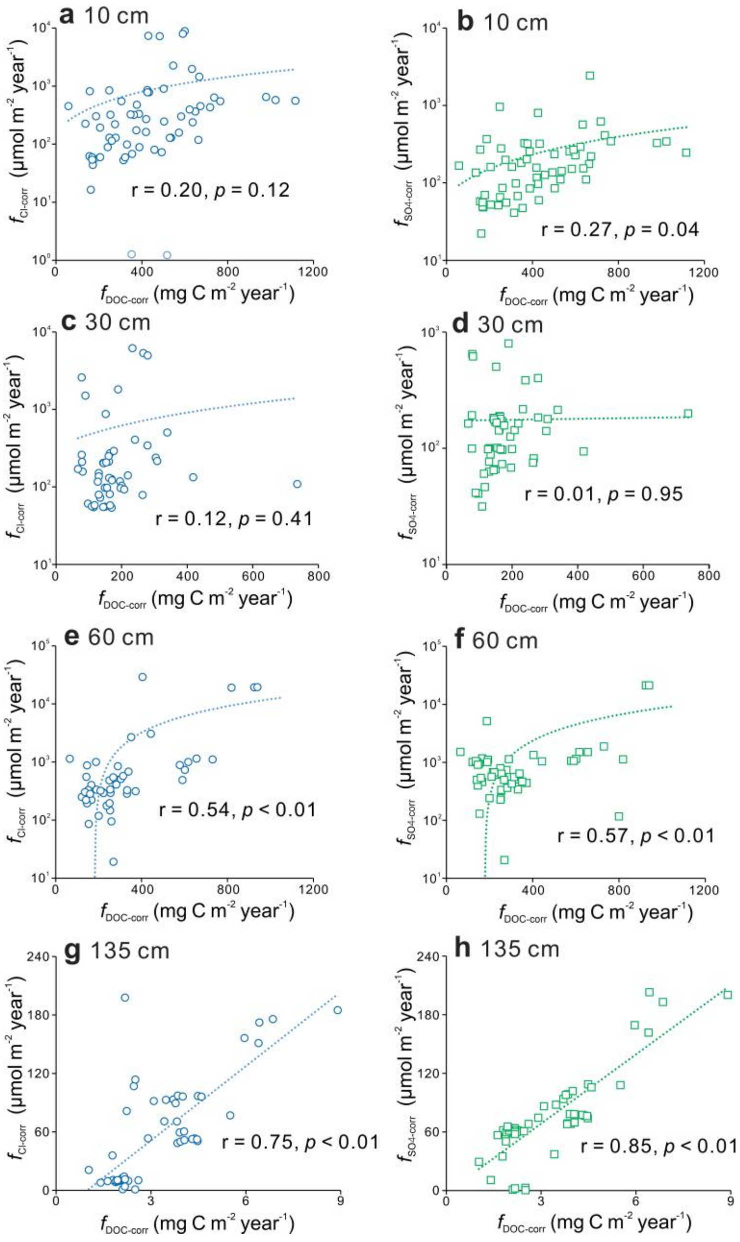

Supplementary Fig. 3 Relationship between corrected dissolved organic carbon (DOC) fluxes (*f*DOC-corr) and corrected fluxes of organic matter decomposition products Cl^- and SO_4^{2-} (*f*Cl-corr and *f*SO4-corr). (a-d) Correlations between $f_{DOC\text{-}corr}$ and $f_{SO4\text{-}corr}$ (and $f_{Cl\text{-}corr}$) are insignificant for topsoil and surface soil (10 cm and 30 cm), whereas (e-h) significant positive correlations are found in midsoil and deep soil. These suggest that production of Cl^- and SO_4^{2-} in the deeper soil with limited nutrients and energy for microorganisms, which may be closely linked to the priming induced by dissolved organic matter input.

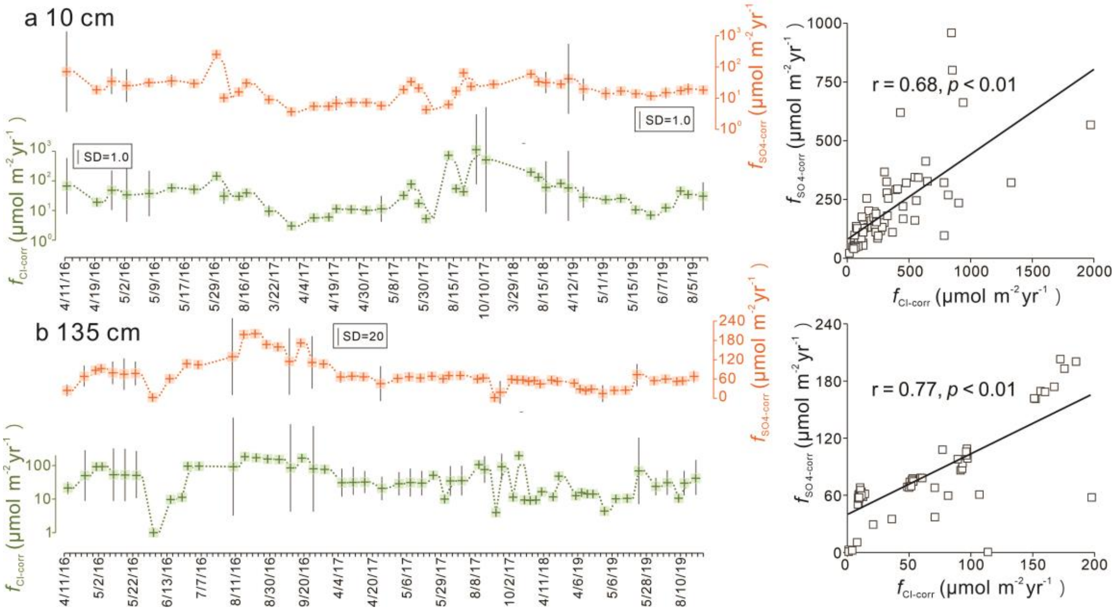

Supplementary Fig. 4 Temporal coupling between corrected fluxes of Cl^- and SO_4^{2-}. Corrected fluxes of Cl^- and SO_4^{2-} ($f_{Cl\text{-}corr}$ and $f_{SO4\text{-}corr}$) show generally consistent time series at 10 cm (a) and 135 cm (b), suggesting that they are both derived from organic decomposition. Each point represents averaged data of two or three measured data from each independent sampling date.

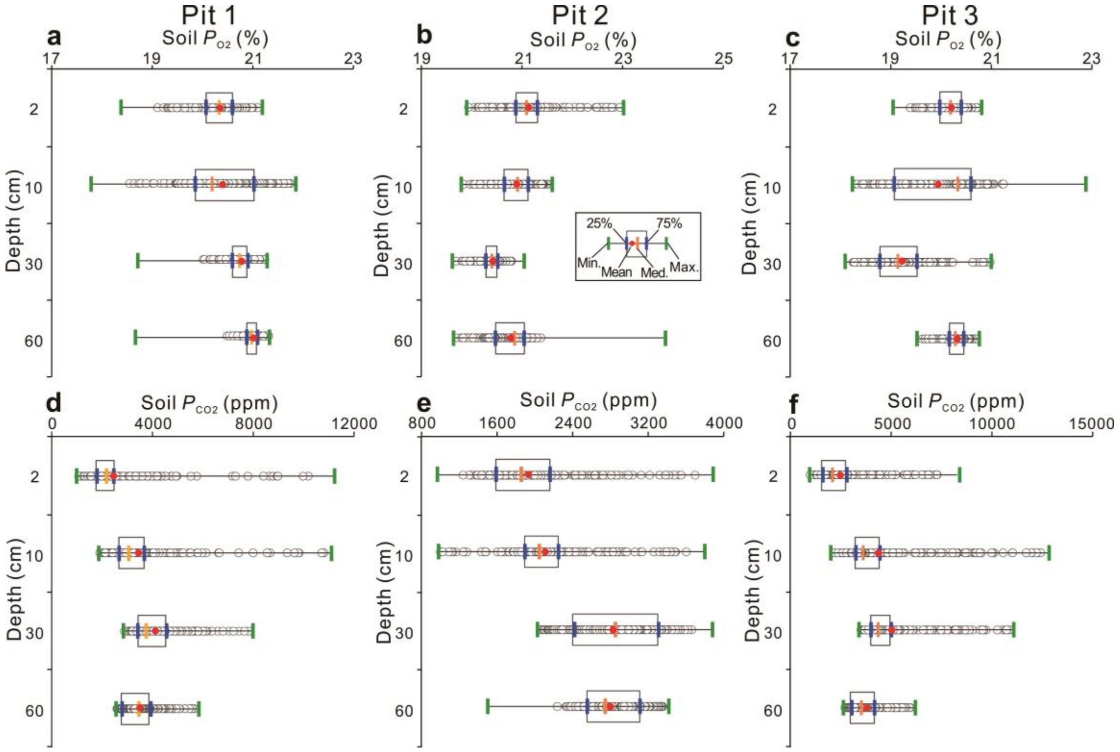

Supplementary Fig. 5 Vertical distributions of soil CO_2 and O_2 partial pressures. (a-c) Box plots of soil O_2 partial pressures (P_{O2}) of the three soil pits. (d-f) Box plots of soil CO_2 partial pressures (P_{CO2}) of the three soil pits. Although we observed soil gas partial pressure at a 15-minute resolution, we did not show each discrete data point (because of too many data) but rather daily-averaged data in these plots. Vertical distribution patterns for partial pressures of a specific gas (O_2 or CO_2) are generally similar for the three soil pits, while vertical patterns for O_2 and CO_2 are generally symmetrical. Soil P_{CO2} or P_{O2} does not linearly change with depth but rather shows maximum or minimum at 30 cm.

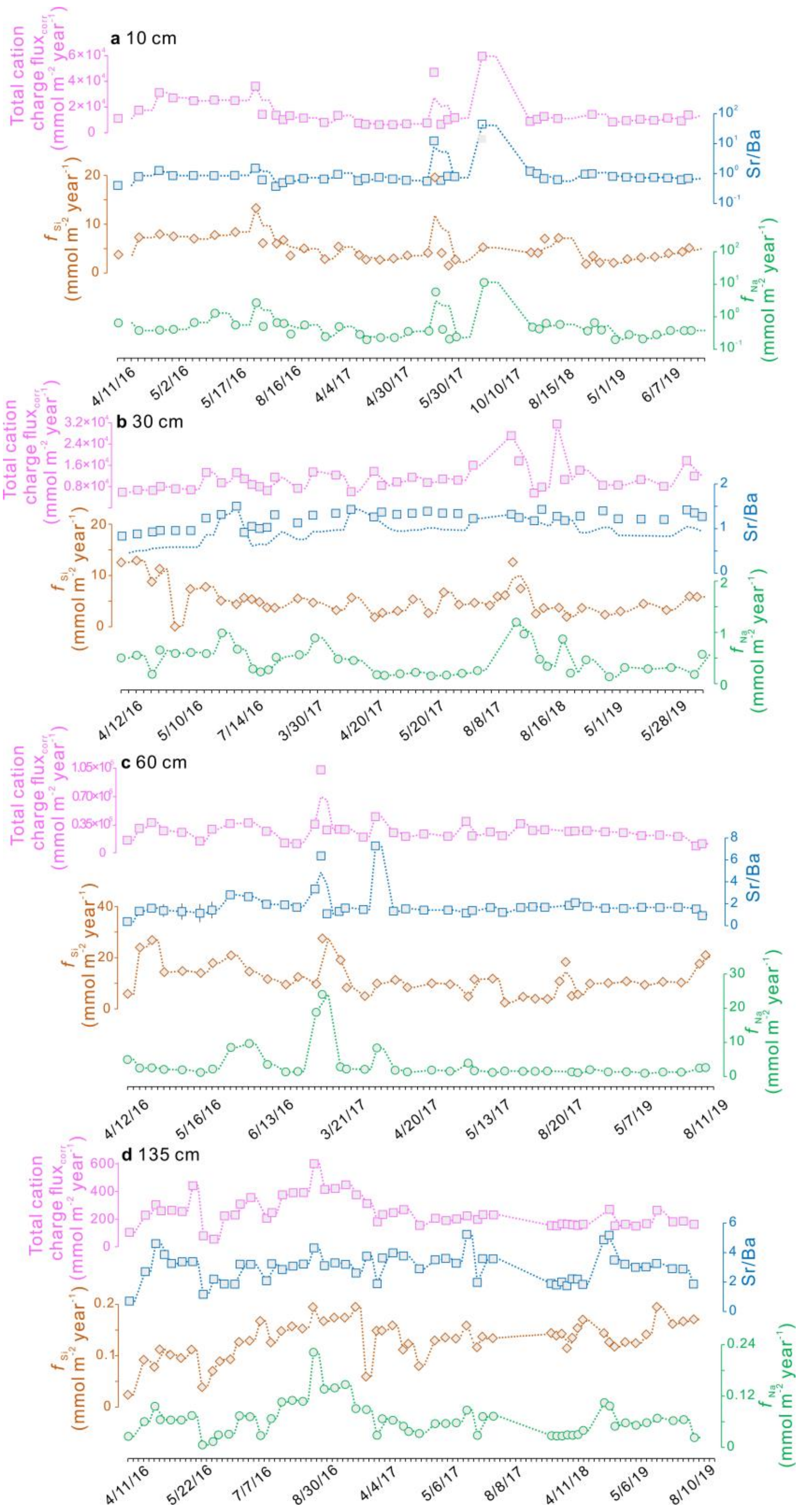

Supplementary Fig. 6 Time series of proxies for mineral weathering intensity. Proxies for weathering intensities including total cation charge flux, Sr/Ba ratio, Si flux (f_{Si}) and Na flux (f_{Na}) at (a) 10 cm, (b) 30 cm, (c) 60 cm, and (d) 135 cm. These proxies show generally consistent temporal trends, suggesting the predominance of silicate weathering in the soil weathering system.

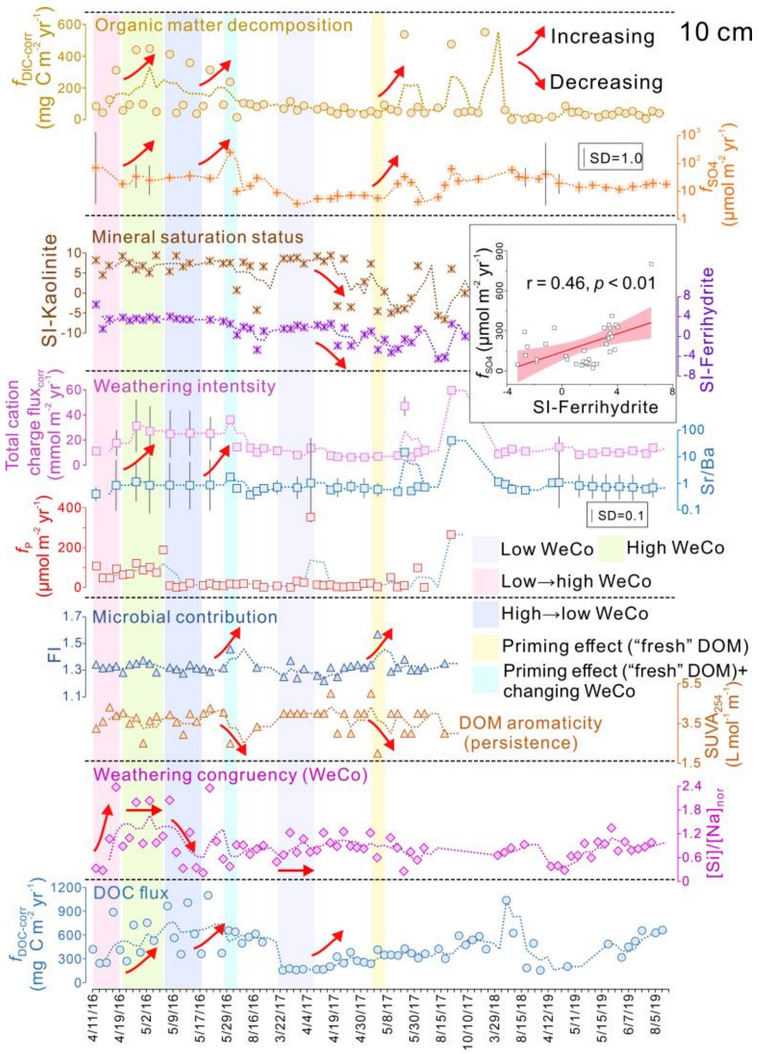

Supplementary Fig. 7 Time series of biogeochemical proxies at 10 cm depth. Proxies for mineral weathering intensity and weathering congruency (WeCo) display a degree of temporal coupling with saturation indices of secondary minerals. These proxies show generally consistent trends with organic matter decomposition rate. Fluorescence index (FI) shows an opposite trend to the parameter that defines dissolved organic matter (DOM) bioavailability. Fluxes of soil organic matter (SOM) decomposition products are similar to the FI time series, suggesting that “primed” microbial activity accelerates organic matter decomposition. The periods with specific biogeochemical characteristics are highlighted with background colors. For example, light purple, light green, pink, and blue bands represent the periods characterized by low, high, low to high, and high to low weathering congruency, respectively, during which specific ultraviolet absorbance at 254 nm ($SUVA_{254}$) and FI were relatively stable. Small red arrows indicate changing trends (increasing or decreasing) in biogeochemical proxies. The light yellow band defines the period with FI increase, $SUVA_{254}$ decrease and little variation in weathering congruency, which can serve as a control group for evaluating the influence of weathering congruency on the priming effect. Abbreviations: corrected dissolved inorganic carbon (DIC) fluxes—$f_{DIC\text{-}corr}$, corrected dissolved organic carbon (DOC) fluxes—$f_{DOC\text{-}corr}$, SO_4^{2-} fluxes—f_{SO4}, saturation index—SI, phosphorous fluxes—f_P, normalized Si concentration/Na concentration—$[Si]/[Na]_{nor}$.

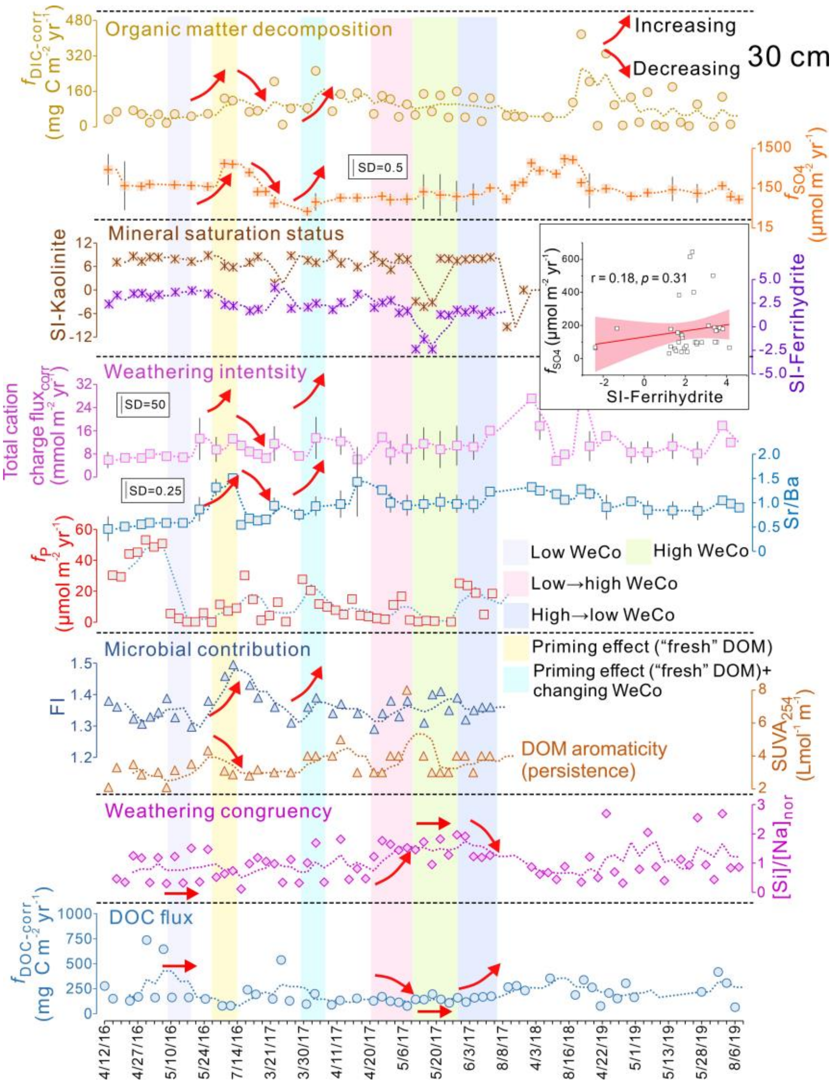

Supplementary Fig. 8 Time series of biogeochemical proxies at 30 cm. Proxies for mineral weathering intensity and weathering congruency display a degree of temporal coupling with saturation indices of secondary minerals. These proxies show generally consistent trends with organic matter decomposition rate. Fluorescence index (FI) shows an opposite trend to the parameter that defines dissolved organic matter (DOM) bioavailability. Fluxes of soil organic matter (SOM) decomposition products are similar to the FI time series, suggesting that "primed" microbial activity accelerates organic matter decomposition. The periods with specific biogeochemical characteristics are highlighted with background colors. For example, light purple, light green, pink, and blue bands represent the periods characterized by low, high, low to high, and high to low weathering congruency, respectively, during which specific ultraviolet absorbance at 254 nm ($SUVA_{254}$) and FI were relatively stable. Small red arrows indicate changing trends (increasing or decreasing) in biogeochemical proxies. The light yellow band defines the period with FI increase, $SUVA_{254}$ decrease and little variation in weathering congruency, which can serve as a control group for evaluating the influence of weathering congruency on the priming effect. Abbreviations: corrected dissolved inorganic carbon (DIC) fluxes—$f_{DIC\text{-}corr}$, corrected dissolved organic carbon (DOC) fluxes—$f_{DOC\text{-}corr}$, SO_4^{2-} fluxes—f_{SO4}, saturation index—SI, phosphorous fluxes—f_P, normalized Si concentration/Na concentration—$[Si]/[Na]_{nor}$.

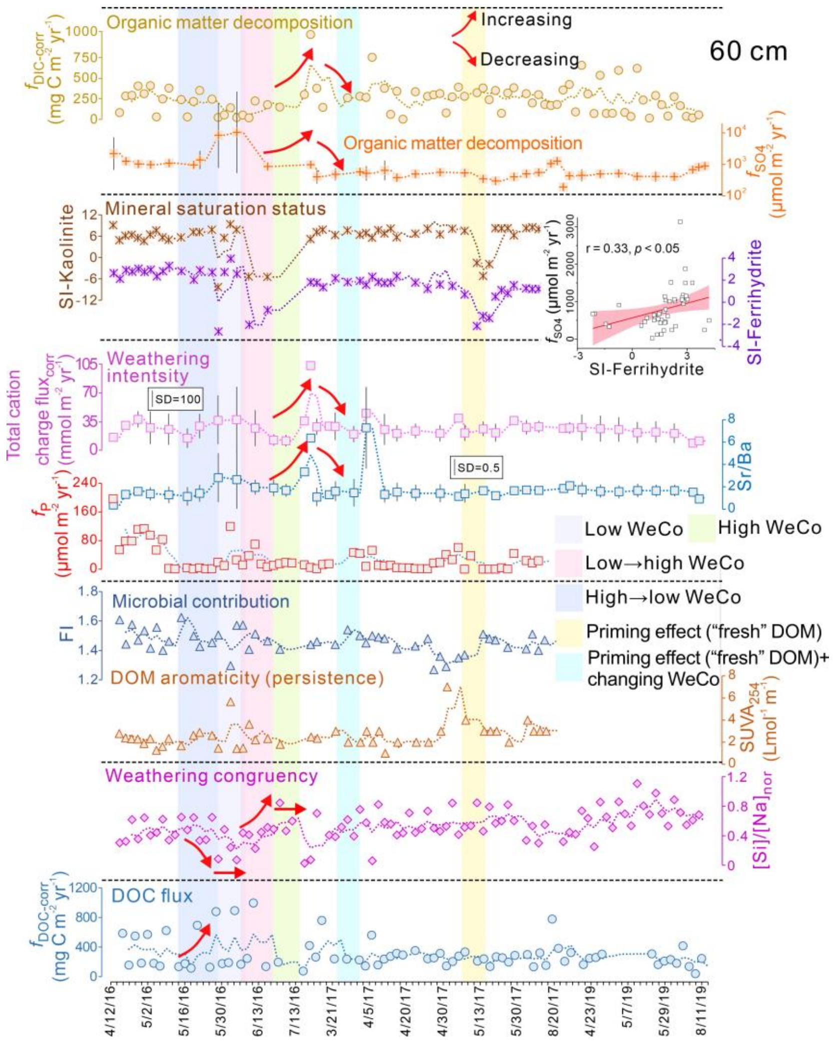

Supplementary Fig. 9 Time series of biogeochemical proxies at 60 cm depth. Proxies for mineral weathering intensity and weathering congruency display a degree of temporal coupling with saturation indices of secondary minerals. These proxies show generally consistent trends with organic matter decomposition rate. Fluorescence index (FI) shows an opposite trend to the parameter that defines dissolved organic matter (DOM) bioavailability. Fluxes of soil organic matter (SOM) decomposition products are similar to the FI time series, suggesting that "primed" microbial activity accelerates organic matter decomposition. The periods with specific biogeochemical characteristics are highlighted with background colors. For example, light purple, light green, pink, and blue bands represent the periods characterized by low, high, low to high, and high to low weathering congruency, respectively, during which specific ultraviolet absorbance at 254 nm ($SUVA_{254}$) and FI were relatively stable. Small red arrows indicate changing trends (increasing or decreasing) in biogeochemical proxies. The light yellow band defines the period with FI increase, $SUVA_{254}$ decrease and little variation in weathering congruency, which can serve as a control group for evaluating the influence of weathering congruency on the priming effect. Abbreviations: corrected dissolved inorganic carbon (DIC) fluxes—$f_{DIC\text{-}corr}$, corrected dissolved organic carbon (DOC) fluxes—$f_{DOC\text{-}corr}$, SO_4^{2-} fluxes—f_{SO4}, saturation index—SI, phosphorous fluxes—f_P, normalized Si concentration/Na concentration—$[Si]/[Na]_{nor}$.

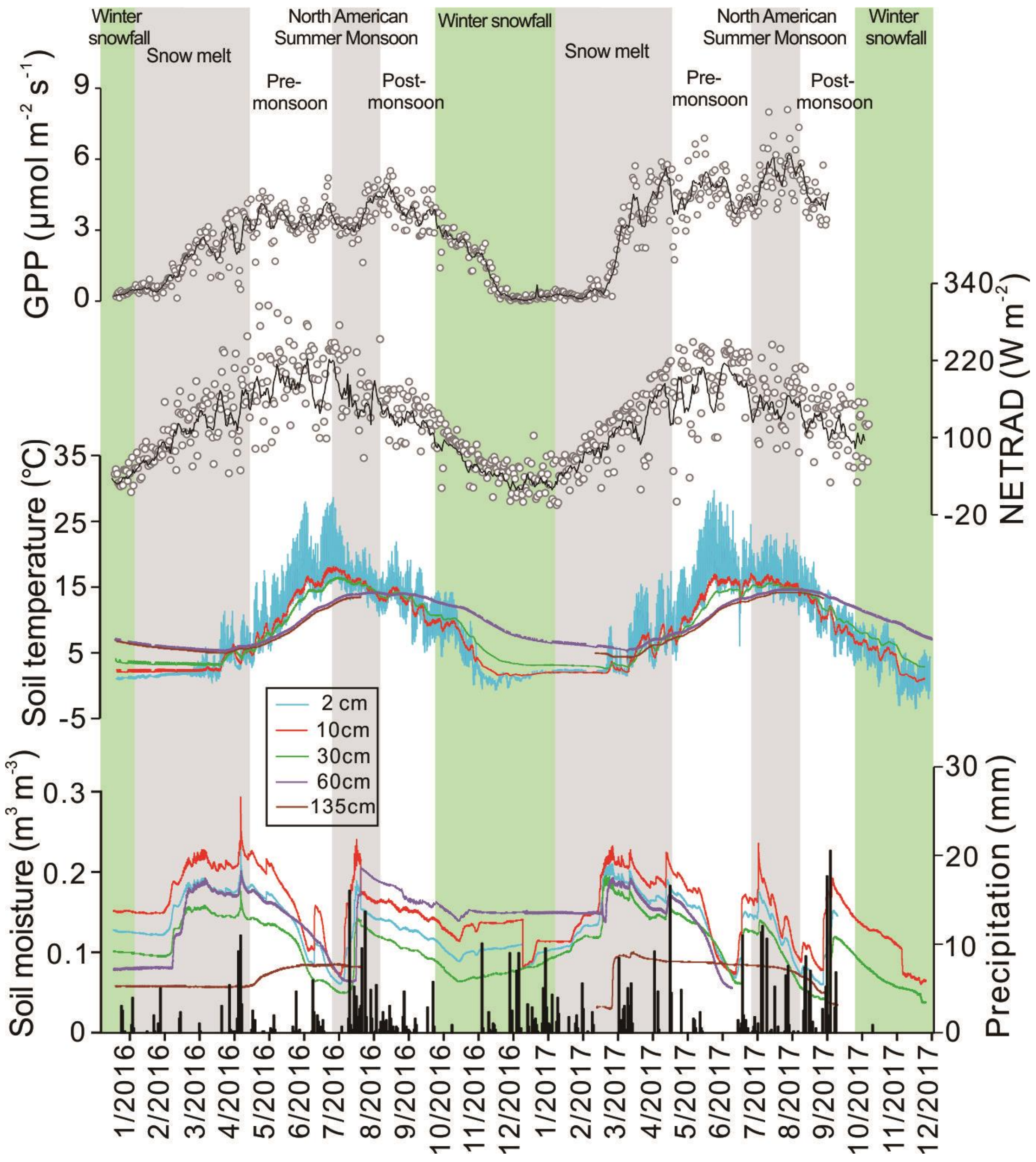

Supplementary Fig. 10 Soil and atmospheric microclimate background. Time-series (1/2016-12/2017) of meteorological background including precipitation, net radiation (NETRAD), gross primary production (GPP), and soil microclimatic parameters including soil temperature and soil moisture at 2cm, 10 cm, 30 cm, 60 cm and 135 cm in the study site (the Sulfur Ridge mixed conifer site, Jemez River Basin Critical Zone Observatory, southwestern US). Also included are precipitation data during this time period. Soil moisture kept relatively high level in spring due to snowmelt input and spring rainfall. Monsoon rainfall led to abrupt increase in soil moisture in summer.

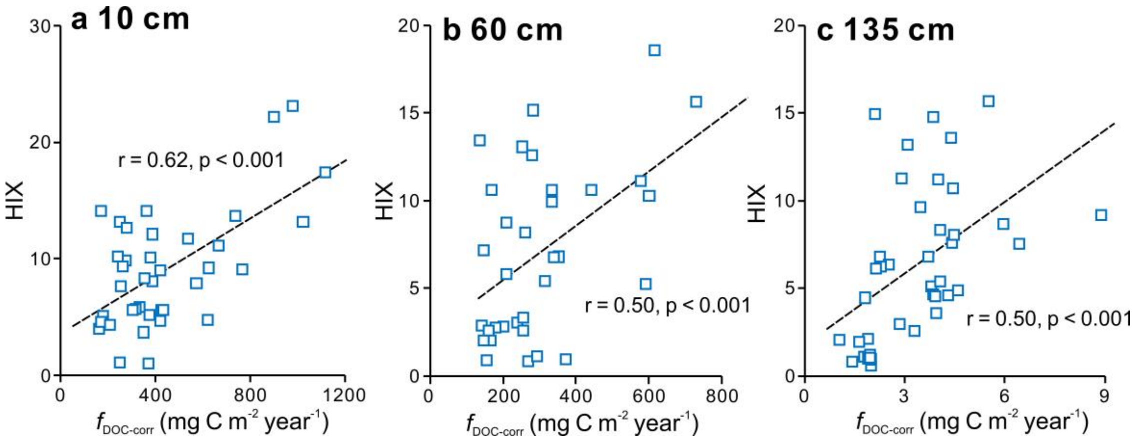

Supplementary Fig. 11 The relationship between corrected dissolved organic carbon (DOC) fluxes ($f_{DOC-corr}$) and humification index (HIX). Significant positive correlations between $f_{DOC-corr}$ and HIX exist at (a) 10 cm, (b) 60 cm and (c) 135 cm. As HIX is obviously higher at shallower depths, the significant correlations may suggest that pulsed dissolved organic carbon flux is potentially associated with dissolved organic matter input from the topsoil.

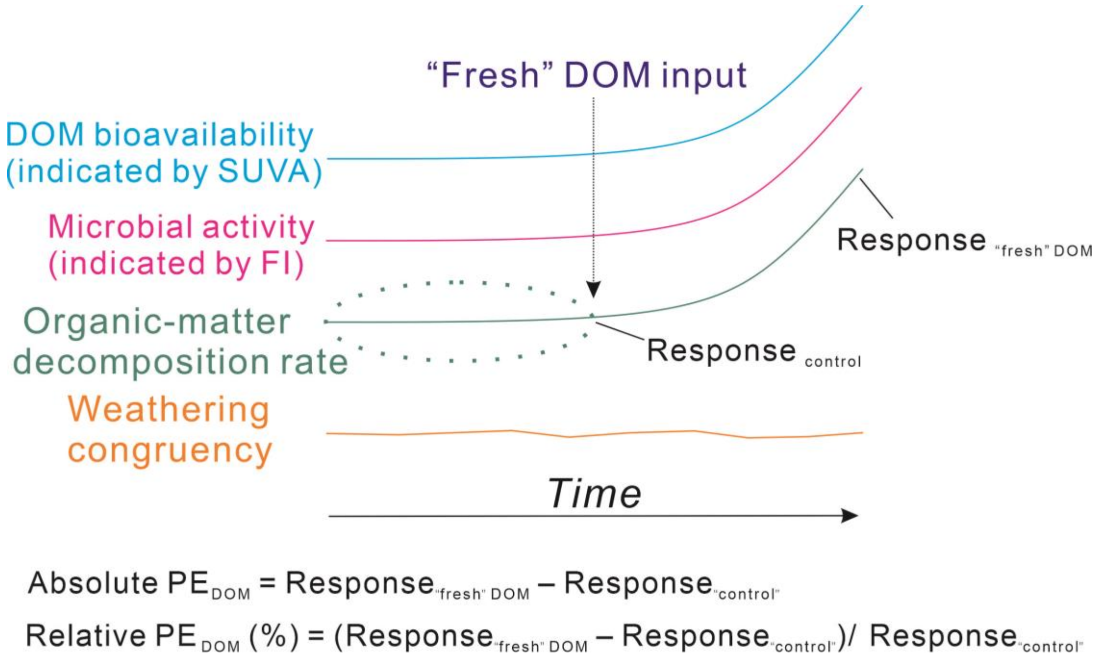

Supplementary Fig. 12 Simplified sketch showing the calculation of priming effect. Organic-matter decomposition rate before the input of "fresh" dissolved organic matter (DOM) is viewed as the calculation control ($\text{Response}_{\text{control}}$), while the increase in decomposition rate after the input of "fresh" DOM can be viewed as priming effect (absolute PE_{DOM}). Abbreviations: dissolved organic matter—DOM, specific ultraviolet absorbance—SUVA, fluorescence index—FI.

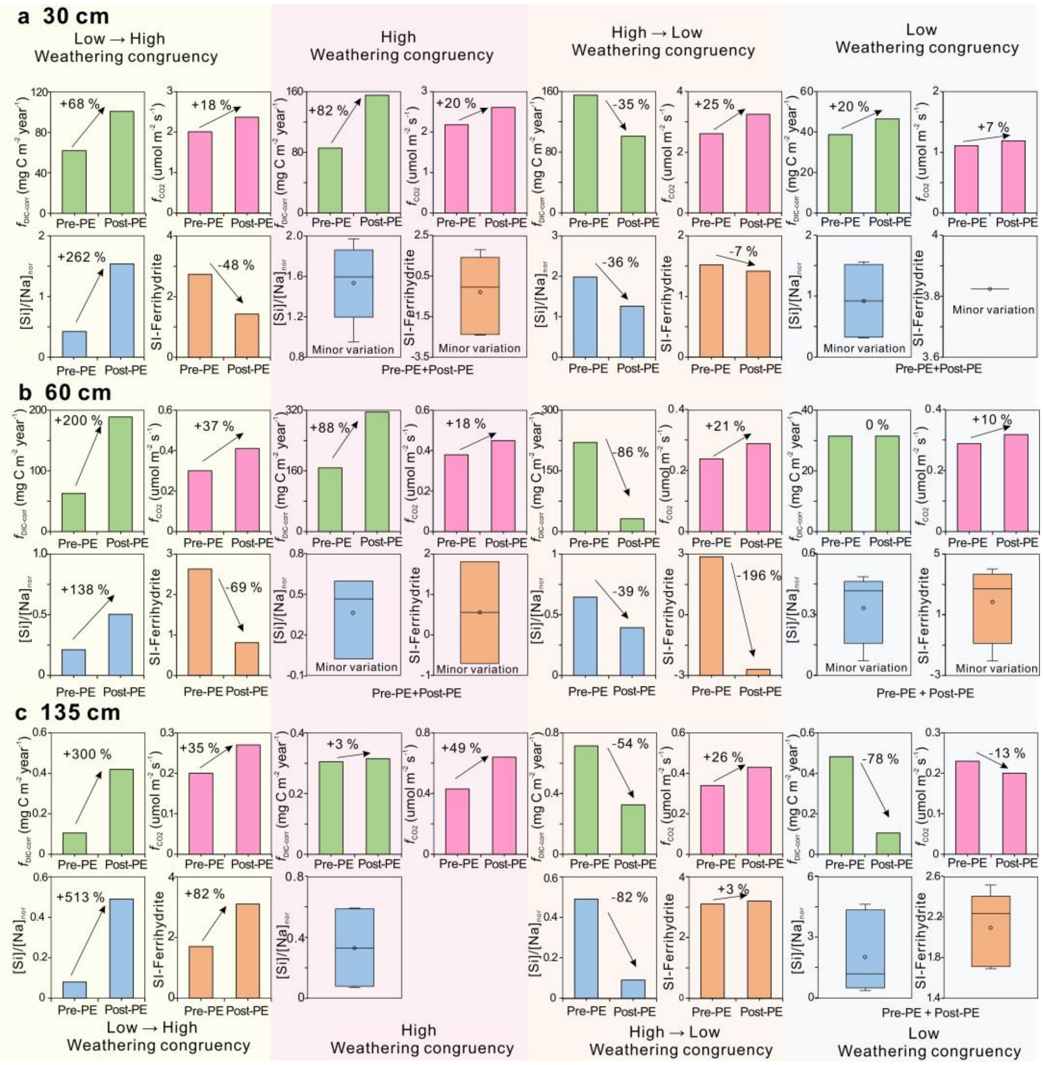

Supplementary Fig. 13 The influence of weathering congruency on organic decomposition rate. This figure shows an indirect evaluation of the linkage between weathering congruency and priming effect (PE) at (a) 30cm, (b) 60 cm and (c) 135 cm. Normalized Si concentration/Na concentration ($[Si]/[Na]_{nor}$) is used to denote weathering congruency with high values denoting high weathering congruency. At each depth, four groups are classified based on weathering congruency, i.e., low to high weathering congruency group, high weathering congruency group, high to low weathering congruency group and low weathering congruency group. In most cases, organic decomposition rate increases with increasing weathering congruency and decreases with diminishing weathering congruency; organic decomposition rate generally increases in high weathering congruency group and decreases in low weathering congruency group. Saturation index (SI)-ferrihydrite is expected to show an opposite variation trend with weathering congruency while the fact may not necessarily be, because saturation status of secondary minerals is determined at least by weathering intensity and weathering congruency. Abbreviations: corrected dissolved inorganic carbon (DIC) fluxes—$f_{DIC\text{-}corr}$, SO_4^{2-} fluxes—f_{SO4}, saturation index—SI, normalized Si concentration/Na concentration—$[Si]/[Na]_{nor}$.

Table S1 General physical and chemical properties of the three soil pedons

Pit number	Sampling depth of porewater	Sampling depth of solid phase	Subdivision	Munsell color	Sand	Silt + Clay	pH	TOC	TN	TIC	Exchangable cations (cmol kg^{-1})				ECEC (cmol kg^{-1})	Al_2O_3	MnO	Fe_2O_3	Al_{PP}	Fe_{PP}	Al_{DC}	Fe_{DC}	Al_{OX}	Fe_{OX}	SSA
	cm	cm			%	%		g kg^{-1}	g kg^{-1}	g kg^{-1}	Na^+	K^+	Ca^{2+}	Mg^{2+}		g kg^{-1}	g kg^{-1}	g kg^{-1}	g kg^{-1}	g kg^{-1}	g kg^{-1}	g kg^{-1}	g kg^{-1}	g kg^{-1}	m^2 g^{-1}
Pit 1	10	5~10	Topsoil	10YR 3/2	52.67	47.28	5.57	10.9	0.6	0	1.29	0.63	2.16	0.28	4.36	133.0	1.3	20.4	0.42	0.33	0.93	3.08	1.09	0.95	1.89
	30	20~30	Surface soil	10YR 5/2	56.2	43.84	5.86	3.9	0.3	0	1.38	0.55	1.45	0.18	3.56	135.8	0.6	20.6	0.37	0.24	0.84	2.65	0.74	0.71	2.44
	60	40~60	Midsoil	10YR 5/2	54.66	45.32	6.09	1.8	0.2	0.2	1.62	0.52	1.43	0.18	3.75	136.9	0.5	19.9	0.36	0.21	0.89	2.71	1.15	1.14	4.84
	135	120~140	Deep soil	2.5Y 6/4	64.78	35.2	5.98	1.2	0.2	0.3	2.20	0.95	2.93	0.17	6.25	218.7	0.8	27.0	0.35	0.18	1.24	2.67	2.17	0.86	27.20
Pit 2	10	5~10	Topsoil	10YR 3/2	45.0	55	5.86	22.0	1.1	0	1.04	0.56	2.86	0.42	4.88	120.8	2.1	20.1	0.45	0.53	0.88	3.38	1.11	1.14	3.42
	30	20~30	Surface soil	10YR 5/4	51.9	48.1	6.02	4.6	0.3	0.1	1.25	0.51	1.14	0.19	3.09	137.7	0.6	18.0	0.43	0.25	0.86	2.91	1.20	0.84	4.08
	60	40~60	Midsoil	2.5Y 7/4	51.8	48.2	6.09	2.1	0.2	0	2.17	1.64	1.43	0.41	5.65	133.0	0.5	21.0	0.37	0.23	0.93	3.46	1.22	1.45	5.87
	135	120~140	Deep soil	2.5Y 6/4	50.9	49.1	6.17	1.2	0.2	0.2	1.90	1.13	2.36	0.60	5.99	215.5	0.8	29.0	0.43	0.19	1.21	3.50	2.75	1.27	30.24
Pit 3	10	5~10	Topsoil	10YR 2/2	48.0	52	5.71	29.4	1.5	0	0.81	0.77	4.01	0.34	5.93	129.0	1.8	20.1	0.65	0.49	0.61	1.06	1.38	1.12	3.84
	30	20~30	Surface soil	10YR 3/2	58.4	41.6	5.95	13.7	0.7	0	1.58	0.62	2.73	0.25	5.18	133.5	1.3	19.4	0.47	0.32	0.59	1.09	1.25	0.86	4.08
	60	40~60	Midsoil	10YR 5/4	64.3	35.7	6.16	6.2	0.3	0.1	0.88	0.65	1.68	0.29	3.50	149.6	0.5	19.7	0.50	0.22	0.56	0.94	1.30	1.05	4.57
	135	120~140	Deep soil	2.5Y 7/4	74.4	25.6	6.28	2.5	0.2	0.1	1.04	0.46	1.36	0.22	3.08	158.3	0.5	16.6	0.50	0.26	0.44	0.08	1.35	0.68	9.94

Abbreviations: TOC: total organic carbon; TN: total nitrogen; TIC: total inorganic carbon; ECEC: effective cation exchange capability; Al_{pp}: content of Al and Fe bound in organometallic complexes (extracted by sodium pyrophosphate); Fe_{pp}: content of Fe bound in organometallic complexes (extracted by sodium pyrophosphate); Al_{DC}: content of Al extracted by dithionite-citrate; Fe_{DC}: content of Fe extracted by dithionite-citrate; Al_{OX}: content of Al extracted by acid ammonium oxalate; Fe_{OX}: content of Fe extracted by acid ammonium oxalate; SSA: specific surface area.

Supplementary Note 1: Soil properties and seasonal variations in water-energy fluxes and soil microclimate

General physical and chemical properties of the three soil pedons are summarized in Supplementary Table 1. The content of clay + silt, which can reflect the contribution of very fine fractions to the soil system, generally decreases with depth. Total organic C (TOC) and N (TN), as well as the C/N ratio, also decline with depth (Supplementary Table 1). TOC shows significant correlations with the contents of Al (Al_{PP}) and Fe (Fe_{PP}) bound in organometallic complexes (extracted by sodium pyrophosphate) but does not show significant correlations with the contents of Al (Al_{OX}) and Fe (Fe_{OX}) of organic complexes, short-range-order Fe (Al)-oxyhydroxides (extracted by acid ammonium oxalate), or the contents of Al (Al_{DC}) and Fe (Fe_{DC}) of organic complexes, which includes reducible “free” iron oxides, including those that are isomorphically substituted with Al (extracted by dithionite-citrate). Soil specific surface area (SSA) increases with depth, ranging from 1.89 to 30.24 m^2 g^{-1}.

Gross primary production (GPP), net radiation (NETRAD), and soil temperature at different depths generally follow similar seasonal patterns, increasing in spring/summer and declining in autumn/winter (Supplementary Fig. 10). This pattern suggests that both GPP and soil temperature are directly controlled by NETRAD. GPP is thought to be closely associated with the interactive influences of air temperature, NETRAD, and soil hydrology. Soil temperature remains stable during January—March and notably increases during April—July. Soil moisture also shows similar seasonal trends with depth, reaching a minimum in summer when high air temperature coincides with low rainfall (Supplementary Fig. 10).

Supplementary Note 2: Homogeneity in source dissolved organic matter (DOM) from different soil depths

DOM can be produced within a soil system (e.g., autochthonous microbial exudates) or can be transported from the surrounding area (e.g., allochthonous terrestrial DOM leached from plant litter and soils). Fluorescence index (FI) higher than 1.55 is generally associated with microbially-derived (autochthonous) DOM, mainly from extracellular release and leachate from land algae/bacteria; terrestrially derived (allochthonous) DOM generally has relatively low FI (< 1.4), sourced from terrestrial plant and soil organic matter (SOM). In this study, the FI values at 10 cm are notably lower compared to other horizons, with values ranging from 1.2 to 1.4, indicating that DOM in the topsoil is mainly terrestrially derived (autochthonous), from decomposition/leaching of plant litter, root and SOM in forest floor layers. This is consistent with the observation that the terrestrial humic-like fluorophore and tryptophan-like fluorophore are enriched at shallower soil depths, as revealed by excitation-emission matrix-parallel factor analysis (Supplementary Fig. 2). At 30 cm, the FI values in summer notably increase but are still less than 1.55 (Supplementary Fig. 8). This observation suggests that microbial activity and contributions to DOM below the topsoil are considerable and sensitive to soil temperature.

FI increases linearly with depth yielding values greater than 1.4-1.5 at 30-135 cm (Fig. 2), suggesting greater enrichment of DOM composition by microbial residues (autochthonous constituents). Excitation-emission matrix-parallel factor analysis also suggests that microbial humic-like and tryptophan-like fluorophores are preferentially enriched in the deep soil (Supplementary Fig. 2), consistent with FI variation. Accordingly, DOM below the topsoil can be supplied by both terrestrial input and microbial release, with increasingly larger microbial contributions with depth (Fig. 2). Vertical variations in DOM sources resemble the patterns reported in previous studies (e.g., ref.[1]). In those studies, DOM in surface soils is characterized by a vegetation-type signature (dominated by lignin-derived phenols and plant-derived carbohydrates), whereas subsoil (>30 cm) DOM is dominated by microbially derived carbohydrates and nitrogen-rich compounds[1]. Downward transport of DOM in soil is accompanied by preferential adsorption of hydrophobic, aromatic compounds (terrestrially derived) by soil mineral components, which can also result in relative enrichment of microbially derived compounds. On the other hand, once introduced into soil, terrestrially derived organic matter is transformed into microbial byproducts, and the resulting DOM may exhibit microbial molecular structure even though it may have originated as terrestrial material[2].

Supplementary References

1. Gabor R. S., Eilers K., McKnight D. M., Fierer N., & Anderson S. P. From the litter layer to the saprolite: Chemical changes in water-soluble soil organic matter and their correlation to microbial community composition. Soil Biology and Biochemistry 68, 166-76 (2014).

2. Lehmann, J. et al. Persistence of soil organic carbon caused by functional complexity. Nature Geoscience 13, 529-534 (2020).
