# Supplementary material for: Mineral weathering is linked to microbial priming in the critical zone
Source: Nat Commun. 2023 Jan 20;14:345. doi: 10.1038/s41467-022-35671-x (PMC9860040; doi:10.1038/s41467-022-35671-x)
Supplement: Supplementary file 2 — Description of Additional Supplementary Files [file 41467_2022_35671_MOESM2_ESM.docx]

**Description of Additional Supplementary Files**

Supplementary Data 1 Soil temperature and moisture at different depths of Pit 1 (2016-2018)

Supplementary Data 2 Soil temperature and moisture at different depths of Pit 2 (2016-2018)

Supplementary Data 3 Soil temperature and moisture at different depths of Pit 3 (2016-2018)

Supplementary Data 4 Soil O_2_ and CO_2_ partial pressures at different depths of Pit 1 (2016-2018)

Supplementary Data 5 Soil O_2_ and CO_2_ partial pressures at different depths of Pit 2 (2016-2018)

Supplementary Data 6 Soil O_2_ and CO_2_ partial pressures at different depths of Pit 3 (2016-2018)

Supplementary Data 7 Soil water chemistry including concentrations of cations, anions and dissolved organic matter, and optical properties of dissolved organic matter (2016, Pit 1-Pit 3)

Supplementary Data 8 Soil water chemistry including concentrations of cations, anions and dissolved organic matter, and optical properties of dissolved organic matter (2017, Pit 1-Pit 3)

Supplementary Data 9 Soil water chemistry including concentrations of cations, anions and dissolved organic matter, and optical properties of dissolved organic matter (2018, Pit 1-Pit 3)

Supplementary Data 10 Soil water chemistry including concentrations of cations, anions and dissolved organic matter, and optical properties of dissolved organic matter (2019, Pit 1-Pit 3)
